# Pharmacokinetic Variability of Direct Oral Anticoagulants and Calcium Channel Blockers: A Comparative Analysis of Exposure Data from Clinical Studies

**DOI:** 10.3390/pharmaceutics18091103

**Published:** 2026-09-02

**Authors:** Lara Marques, Nuno Vale

**Affiliations:** 1 PerMed Research Group, RISE-Health, Faculty of Medicine, University of Porto, Alameda Professor Hernâni Monteiro, 4200-319 Porto, Portugal; lfmarques@med.up.pt; 2 RISE-Health, Department of Community Medicine, Health Information and Decision (MEDCIDS), Faculty of Medicine, University of Porto, Rua Doutor Plácido da Costa, 4200-450 Porto, Portugal; 3 Laboratory of Personalized Medicine, Department of Community Medicine, Health Information and Decision (MEDCIDS), Faculty of Medicine, University of Porto, Rua Doutor Plácido da Costa, 4200-450 Porto, Portugal

**Keywords:** pharmacokinetic variability, direct oral anticoagulants, calcium channel blockers, drug exposure, precision dosing, therapeutic drug monitoring

## Abstract

**Background/Objectives:** Interindividual pharmacokinetic (PK) variability remains a daunting challenge for effective and safe drug therapy. Despite the widespread use of direct oral anticoagulants (DOACs) and calcium channel blockers (CCBs), a substantial number of adverse drug reactions have been reported for both classes. Herein, this study aimed to assess and analyze the PK variability of DOACs and CCBs across diverse clinical and demographic profiles under both single- and multiple-dose conditions. **Methods:** A PubMed search identified clinical PK studies reporting maximum plasma concentration (C_max_) and/or area under the concentration-time curve (AUC). The coefficient of variation (CV%) was calculated and used as a measure of PK variability. A CV% < 40% indicated low-to-moderate variability, and a CV% > 40% was defined as high variability. **Results:** A total of 264 studies were included following systematic screening, and the dataset was further characterized according to population features and clinical context. Among DOACs, edoxaban exhibited the lowest PK variability, whereas dabigatran showed the highest. CCBs demonstrated a broad variability spectrum, ranging from predictable agents (amlodipine and felodipine) to highly variable compounds (nisoldipine, isradipine, nimodipine, diltiazem, and verapamil). Studies evaluating drug–drug interactions, ethnicity, and specific drug-related factors were associated with increased PK variability. **Conclusions:** These findings suggest that fixed-dose strategies may not be universally appropriate for DOACs and CCBs, particularly in high-risk subgroups where altered exposure may lead to sub- or supratherapeutic concentrations and compromise clinical outcomes. Therefore, clinicians should avoid evaluating individual risk factors in isolation and instead consider the patient’s complete profile when selecting and adjusting pharmacotherapy.

## 1. Introduction

“Does one dose fit all?” has become one of the most relevant and widely debated questions in modern medicine. Any discussion surrounding the “one-size-fits-all” paradigm inevitably leads to the concept of pharmacokinetic (PK) variability. In clinical practice, PK variability represents a daunting challenge to effective drug therapy [1,2,3]. A dose proven therapeutically effective in some patients may be ineffective or even toxic in others. Variability in therapeutic response among patients with the same clinical condition arises from a wide range of sources of interindividual variability (IIV). These include physiological factors such as age, sex, genetic makeup, pregnancy, diet, microbiome; pathological factors including disease state, obesity, hepatic or renal impairment; and pharmacological factors such as drug–drug interactions (DDIs) and biopharmaceutical formulations [1,3]. Together, these factors influence the processes of absorption, distribution, metabolism, and excretion (ADME), resulting in substantial differences in systemic exposure following administration of a standard dose.

Clinical practice routinely demonstrates considerable differences in plasma drug concentrations between patients, and dose adjustment is often performed through a trial-and-error approach [1,2]. Growing recognition that patients do not respond uniformly to the same treatment has driven the development of tools capable of predicting drug disposition and identifying potential PK liabilities, including poor bioavailability, altered hepatic or renal clearance, susceptibility to DDIs, and the need for dose adjustment in specific patient populations [1,4,5]. Advances in pharmacogenomics, pharmacometrics, and therapeutic drug monitoring (TDM) have improved clinicians’ ability to individualize therapy, supporting the goal of administering the right drug at the right dose to the right patient at the right time. Consequently, identifying and mitigating sources of IIV, such as intestinal permeability, plasma protein binding, first-pass metabolism, and the degree of renal impairment, is essential for optimizing drug safety and efficacy.

The present study focuses on the interindividual PK variability of direct oral anticoagulants (DOACs) and calcium channel blockers (CCBs).

**Figure 1:**
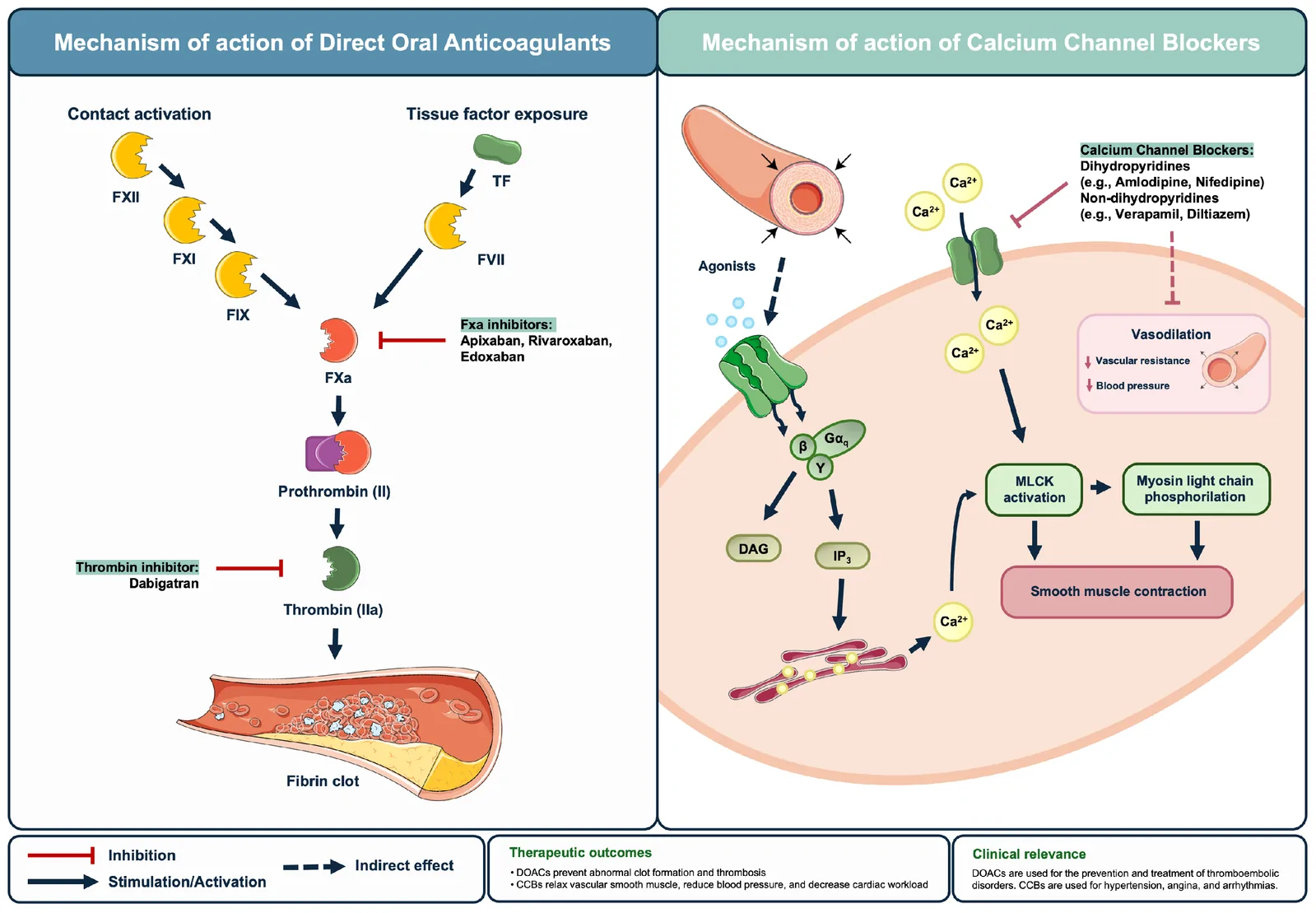
Overview of the mechanisms of action of DOACs and CCBs. DOACs inhibit key steps of the coagulation cascade, thereby reducing thrombin generation and fibrin clot formation. Factor Xa inhibitors (apixaban, rivaroxaban, and edoxaban) block the conversion of prothrombin to thrombin, while dabigatran directly inhibits thrombin. CCBs inhibit L-type calcium channels in vascular smooth muscle and cardiac tissue, reducing intracellular calcium influx, decreasing smooth muscle contraction, promoting vasodilation, and lowering blood pressure. Dihydropyridines (nifedipine, amlodipine, nicardipine, felodipine, and nimodipine, etc.) primarily exert vascular effects, while non-dihydropyridines (diltiazem and verapamil) additionally reduce cardiac conduction and contractility. Created with Servier Medical Art (https://smart.servier.com), licensed under CC BY 4.0 (https://creativecommons.org/licenses/by/4.0/) (accessed on 10 June 2026).

DOACs are widely prescribed for the prevention of venous thromboembolism (VTE) and stroke in patients with atrial fibrillation (AF) [5,6], as they interrupt part of the complex system involved in the formation of blood clots (Figure 1) [7]. This drug class comprises two main categories: direct thrombin inhibitors (dabigatran) and direct factor Xa inhibitors (rivaroxaban, apixaban, and edoxaban) [6,8,9]. Following their first approval by the U.S. Food and Drug Administration (FDA) in 2010, DOACs have rapidly become reliable alternatives to vitamin K antagonists (VKAs), which had long represented the standard of care (SoC) for oral anticoagulation [6]. Their widespread adoption was owing to several advantageous properties: improved patient adherence due to oral administration, rapid onset of action, fixed-dose regimens, limited IIV with predictable PK and pharmacodynamic (PD) profiles, reduced risks of food and drug interactions, and a lower incidence of adverse events, particularly bleeding complications [10,11,12].

Since their introduction, DOACs have remained a controversial topic in routine clinical practice regarding whether therapeutic monitoring is necessary [13]. Some authors argue that routine monitoring is not required because of their predictable PK/PD characteristics [5,6]. However, other studies have shown that approximately 25% of patients receiving DOAC therapy are incorrectly dosed [14,15]. Furthermore, DOACs have been associated with considerable variability in systemic drug exposure, which may increase the risk of adverse clinical outcomes such as bleeding or thromboembolic events [13]. Indeed, a total of 268,551 adverse drug reactions (ADRs) have been reported for DOACs [8]. This retrospective analysis of ADRs associated with DOAC dosing errors demonstrated that underdosing remains the most frequent dosing error, in contrast to overdosing. In addition, a study based on data from the Portuguese pharmacovigilance system between 2012 and 2021 revealed that dabigatran was the anticoagulant most frequently associated with ADRs leading to hospitalization, followed by rivaroxaban, apixaban, and edoxaban [16]. Data from EudraVigilance also showed that rivaroxaban is the oral anticoagulant most frequently reported in ADR cases [17]. Therefore, while some cases of inappropriate dosing may result from a lack of awareness of DOAC prescribing recommendations [6,13], particular population subgroups, including patients with renal/hepatic impairment, obesity, and cancer-associated thrombosis, may represent potential candidates for DOAC monitoring due to the possibility of exhibiting altered PK profiles [6].

In turn, CCBs represent a heterogeneous group of compounds that primarily act by inhibiting the influx of calcium ions across the cell membrane through blockade of L-type voltage-gated calcium channels in the myocardium, vascular smooth muscle, and pancreas (Figure 1) [18,19]. Within this class, CCBs can be classified as non-dihydropyridine (non-DHP) CCBs—diltiazem and verapamil—which are cardioselective and exert their effects predominantly on the myocardium, and dihydropyridine (DHP) CCBs—nifedipine, amlodipine, nicardipine, felodipine, and nimodipine, among others –which primarily act on vascular smooth muscle [18,20]. Their therapeutic indications include hypertension, supraventricular tachycardia, vasospasm, and migraine headaches [21,22,23]. Several PK studies have reported high IIV with CCB exposure (Table 1), raising concerns about their long-term safety and efficacy [24]. In fact, these drugs have been associated with adverse outcomes, such as increased risk of new-onset diabetes [25], impaired renal safety profiles [26], and bone fractures [27]. In addition, clinical manifestations such as bradycardia and hypotension have been reported [21,28].

According to the 2024 report of the American Association of Poison Control Centers’ National Poison Data System (NPDS), 6693 single exposures to CCBs were reported in the United States, of which 39 were associated with fatalities, and 1166 occurred in children aged five years or younger [28]. The increasing therapeutic use of these drugs likely contributes to these elevated numbers. Isbister et al. [37] have identified amlodipine, diltiazem, and verapamil as the most toxic agents within the CCB class. A systematic review demonstrated that, although CCBs remain acceptable options for long-term therapy, monitoring may be necessary, particularly in high-risk populations, due to their toxicity [24]. Furthermore, most of the supporting evidence is derived from studies involving DHP CCBs, highlighting the limited number of studies available for non-DHP CCBs.

The reported toxic effects may, at least in part, result from PK variability that is not adequately managed at the time of prescribing. Similarly, treatment failure may indicate that external factors are affecting the disposition of these compounds, leading to suboptimal drug exposure. Therefore, a comprehensive understanding of the sources of IIV represents a critical step towards achieving optimal dose selection, thereby maximizing therapeutic efficacy while minimizing unnecessary exposure to supratherapeutic drug concentrations. As far as we know, this is the first systematic comparative analysis to comprehensively characterize and quantify the PK variability of these two drug classes. Previous evidence has generally focused on individual agents or selected patient populations, limiting direct comparisons of the magnitude and consistency of PK variability across drugs and clinical scenarios. The lack of consensus on the true extent of variability in systemic exposure to DOACs and CCBs may introduce uncertainty into clinical decision-making, helping explain the frequent occurrence of inappropriate underdosing in DOAC-treated patients. By integrating data from single-dose and steady-state studies and from populations with diverse demographic and clinical characteristics, this study provides a broader assessment of the determinants and clinical patterns of variability in systemic exposure. This work provides a quantitative framework for identifying situations in which conventional dosing may be associated with greater uncertainty in systemic exposure, providing guidance for safer and more personalized prescribing strategies in clinical practice.

## 2. Methods

The PubMed database was used to identify PK studies involving four DOACs (apixaban, rivaroxaban, edoxaban, and dabigatran) and 11 CCBs (amlodipine, felodipine, nifedipine, nicardipine, nisoldipine, isradipine, nimodipine, lecarnidipine, diltiazem, and verapamil) commonly prescribed for patients with cardiovascular diseases. The literature search included studies published up to April 2026. For each drug, the following search strategies were applied: “drug name AND pharmacokinetics AND single dose” for single-dose (SD) studies, and “drug name AND pharmacokinetics AND (steady state OR multiple dose)” for steady-state (SS) studies.

SD and SS studies conducted in healthy adults and pediatric populations, as well as in patients with renal or hepatic impairment, and studies evaluating the concomitant administration of a DOAC or CCB with another drug, were included for variability analysis. PK results describing drug exposure were collected as the coefficient of variation (CV), using the most appropriate PK parameters, namely maximum plasma concentration (C_max_) and area under the concentration-time curve (AUC). Studies reporting only mean values or individual data presented exclusively in graphical format were excluded, as CV values could not be calculated. Each PK observation was defined as a unique combination of study, PK parameter (Cmax or AUC), dosing regimen (single-dose or steady-state), dose level, and study population or clinical scenario (e.g., DDI, renal impairment, ethnicity, or age). Multiple observations extracted from the same publication were retained only when they represented distinct experimental conditions and were therefore considered independent PK contexts rather than repeated measurements of the same dataset.

Variability data for PK parameters were reported in different formats, including mean with CV%, mean with standard deviation (SD), or mean with standard error of the mean (SEM). Both SD and SEM were converted into CV% when necessary. For studies reporting SD, CV was calculated using the formula: CV=SDMean×100. For studies reporting SEM, SD was back-calculated using the following equation, where *n* represents the number of study participants: $SD=SEM\times \sqrt{n}$. The calculated SD value was subsequently used to derive CV%.

Descriptive statistics were used to summarize PK variability (CV%) for C_max_ and AUC across individual drugs, drug classes, study designs, and study populations. For both C_max_ and AUC, the CV reported for each study (SD or SS) was classified into the following categories: 0–20%, 20–40%, 40–60%, 60–80%, or >80%. For descriptive interpretation, studies with CV values > 40% were considered to exhibit high PK variability, whereas those with CV values < 40% were considered to demonstrate low-to-moderate PK variability. This threshold was selected as a pragmatic criterion to facilitate comparisons across compounds and clinical scenarios.

Differences in the distribution of CV values between individual drugs within each drug class were assessed separately for C_max_ and AUC and according to study design (SD and SS) using the Kruskal–Wallis test. Post-hoc pairwise comparisons were performed using Dunn’s test with Holm adjustment for multiple comparisons. Differences in PK variability across population categories were also assessed separately within DOACs and CCBs using the Kruskal–Wallis test. To further evaluate factors associated with PK variability after adjustment for potential confounding variables, multivariable linear regression models were developed. Because CV values showed a right-skewed distribution, CV% were log-transformed before regression analysis. Drug, study design, and study population were included as explanatory variables. Apixaban and amlodipine were specified as the reference drugs for the DOAC and CCB models, respectively, while healthy populations and SD studies were used as reference categories. Regression coefficients (β), 95% confidence intervals, and corresponding adjusted effects [exp(β)] were estimated. Statistical significance was defined as a two-sided *p*-value < 0.05.

Data analysis was performed using R (Version 2026.04.0+526). Generative artificial intelligence (GenAI) tools were employed as supportive resources for manuscript drafting, language refinement, and coding assistance throughout the analytical workflow. GenAI tools were not involved in data generation, autonomous data analysis, or scientific decision-making.

## 3. Results

**Figure 2:**
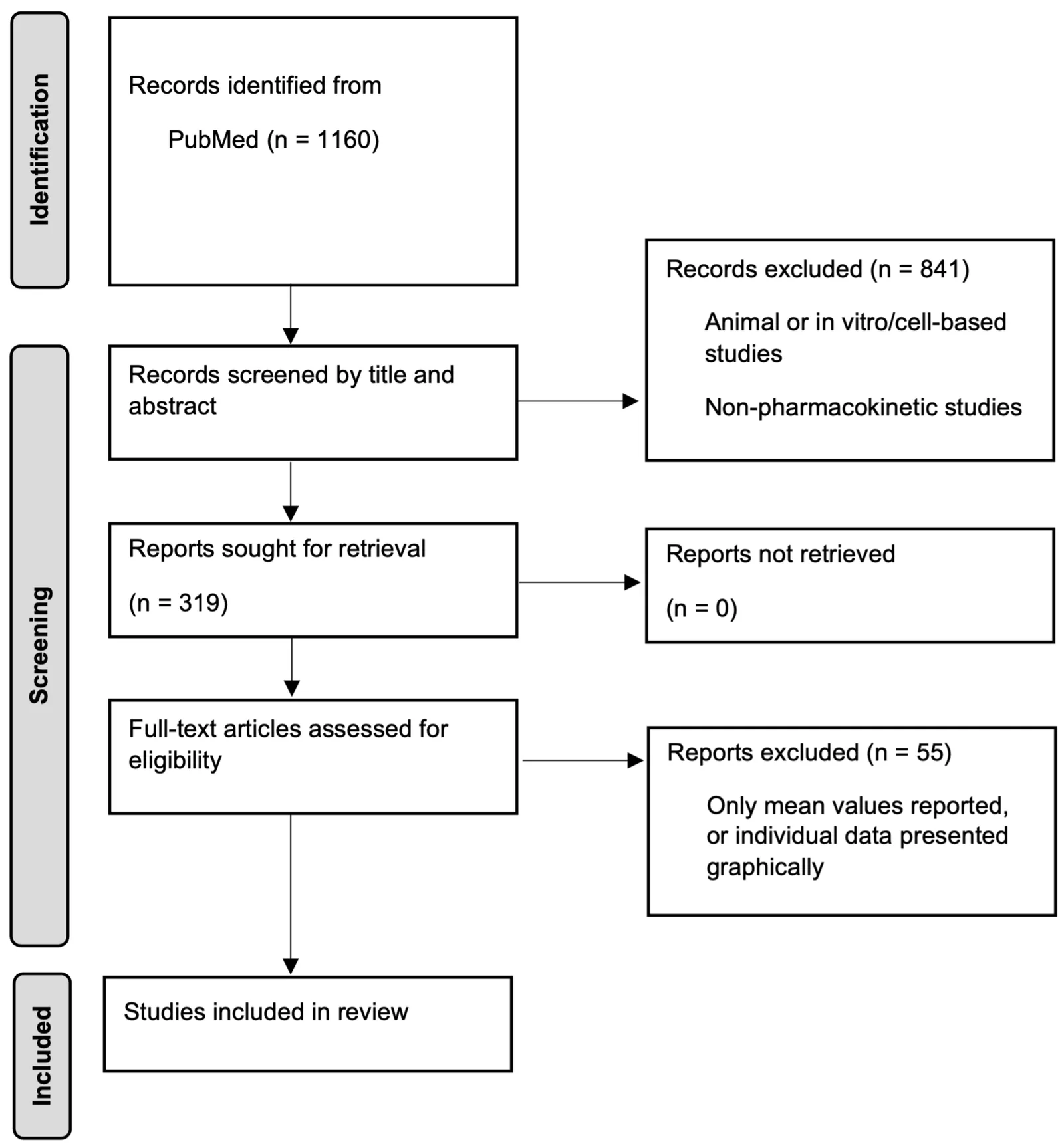
Study selection process. The literature search identified 1160 records. Following title and abstract screening, 319 articles were selected for full-text assessment. 264 studies were included in the final analysis after evaluation against the pre-defined eligibility criteria. The included studies investigated the DOACs apixaban, rivaroxaban, edoxaban, and dabigatran, as well as the CCBs amlodipine, felodipine, nifedipine, nicardipine, nisoldipine, isradipine, nimodipine, diltiazem, and verapamil.

A total of 319 studies matching the predefined search criteria were initially identified. Following retrieval, 264 studies were included in the final analysis, and 55 studies were excluded because the reported PK parameters could not be converted into CV values (Figure 2). The final study library comprised four DOACs and ten CCBs, although 11 CCBs were initially screened. No eligible PK studies involving lercanidipine were identified, and this compound was therefore excluded from the analysis.

Specifically, 15 SD and SS studies were identified for apixaban, 25 for rivaroxaban, 11 for edoxaban, and 26 for dabigatran. The number of extracted PK observations varied substantially between compounds. Rivaroxaban contributed to the largest number of AUC observations in SD studies (*n* = 98), while edoxaban contributed the fewest (*n* = 28). Among the CCBs, amlodipine generated the largest number of C_max_ and AUC observations, though nicardipine was represented by the smallest number of studies and PK observations. A detailed summary of observations extracted from SD and SS studies is presented in Table 2.

### 3.1. Characterization of PK Variability Across DOACs and CCBs

PK variability was subsequently categorized into five CV ranges. As shown in Table 3, most DOAC data were concentrated within the 20–40% CV category. Apixaban exhibited predominantly low variability, with most observations falling within the 20–40% range. Only 16 measurements exceeded 40% CV for both C_max_ and AUC in SD studies, out of a total of 95 observations. Rivaroxaban also demonstrated a generally low variability profile. In SD studies, only a limited number of measurements fell within the 40–60% CV range, with the majority of values distributed within lower variability categories. Equally, edoxaban displayed a highly stable PK profile, with very few observations indicating moderate-to-high variability. Only six SD measurements were classified within the 40–60% CV range, and no SS studies reported CV values exceeding 40% for C_max_. In contrast, dabigatran showed the highest degree of variability among DOACs. When comparing SD and SS conditions, a general trend towards reduced variability was observed across all drugs. For instance, apixaban showed fewer observations exceeding 40% CV under SS conditions compared with SD, indicating improved exposure consistency during chronic administration.

Table 4 extends the previous analysis to CCBs. Among DHP CCBs, amlodipine and felodipine generally unveiled lower and more predictable variability profiles. Amlodipine was predominantly associated with CV values within the 20–40% range. Felodipine also showed mainly low variability, although some studies reported CV values exceeding 40% and 60% in SD conditions. In contrast, nifedipine displayed a highly heterogeneous PK profile, with CV values distributed across nearly all variability categories for both C_max_ and AUC. Additionally, some DHPs demonstrated marked PK variability: nisoldipine, based on a limited dataset of seven studies, consistently showed high variability across the available evidence; isradipine, evaluated in four studies, also exhibited predominantly moderate-to-high variability; nicardipine was likewise included in a limited number of studies (*n* = 3), two of which reported low variability, while one study under SS conditions showed high variability; and nimodipine showed substantial heterogeneity, with CV values spanning from low to high ranges, including a substantial number of studies reporting values above 40%, indicating prominent variability.

Within non-DHP CCBs, diltiazem revealed a broadly heterogeneous distribution of CV values, with some studies reporting variability above 80%. Verapamil also showed a heterogeneous PK profile; however, a greater proportion of studies reported lower variability. Despite this, several measurements exceeded 40% CV, including four observations above 80%, indicating the presence of clinically relevant variability in specific contexts. Based on our results, CCBs can be stratified into three categories: (i) relatively stable compounds—amlodipine, felodipine, and nicardipine; (ii) highly heterogeneous compounds, for which behavior is difficult to predict in patients—nifedipine, nimodipine, diltiazem, and verapamil; and (iii) compounds associated with high PK variability, namely nisoldipine and isradipine.

Table 5 focuses on the clinical risk threshold, detailing the proportion of PK studies that reported a CV > 40% for AUC, thereby indicating high variability in total drug exposure. According to these results, edoxaban and nicardipine were not associated with highly variable AUC observations under either SD or SS conditions. Amlodipine also showed a low proportion of studies exceeding this threshold. In contrast, nisoldipine, isradipine, nimodipine, diltiazem, and verapamil were more frequently associated with CV values above 40%. One of the main strengths of this analysis is its ability to identify compounds for which high variability represents a common characteristic rather than an isolated observation. Hence, nisoldipine, isradipine, and nimodipine were again flagged as compounds of particular clinical concern. Furthermore, this analysis highlights situations in which multiple-dose administration may improve patients’ outcomes. For dabigatran, the proportion of highly variable studies decreased by 20% when comparing SD and SS conditions. Similarly, for rivaroxaban, the proportion of studies with CV > 40% decreased from 29% (SD) to 20% (SS). The most striking example was observed for nimodipine, where all SD studies were classified as highly variable, while only 17% of SS studies exceeded the 40% CV threshold, suggesting a substantial improvement in exposure consistency following repeated administration. Nevertheless, as the available evidence was not evenly distributed across compounds under SD and SS conditions, these comparisons should be interpreted with caution.

**Figure 3:**
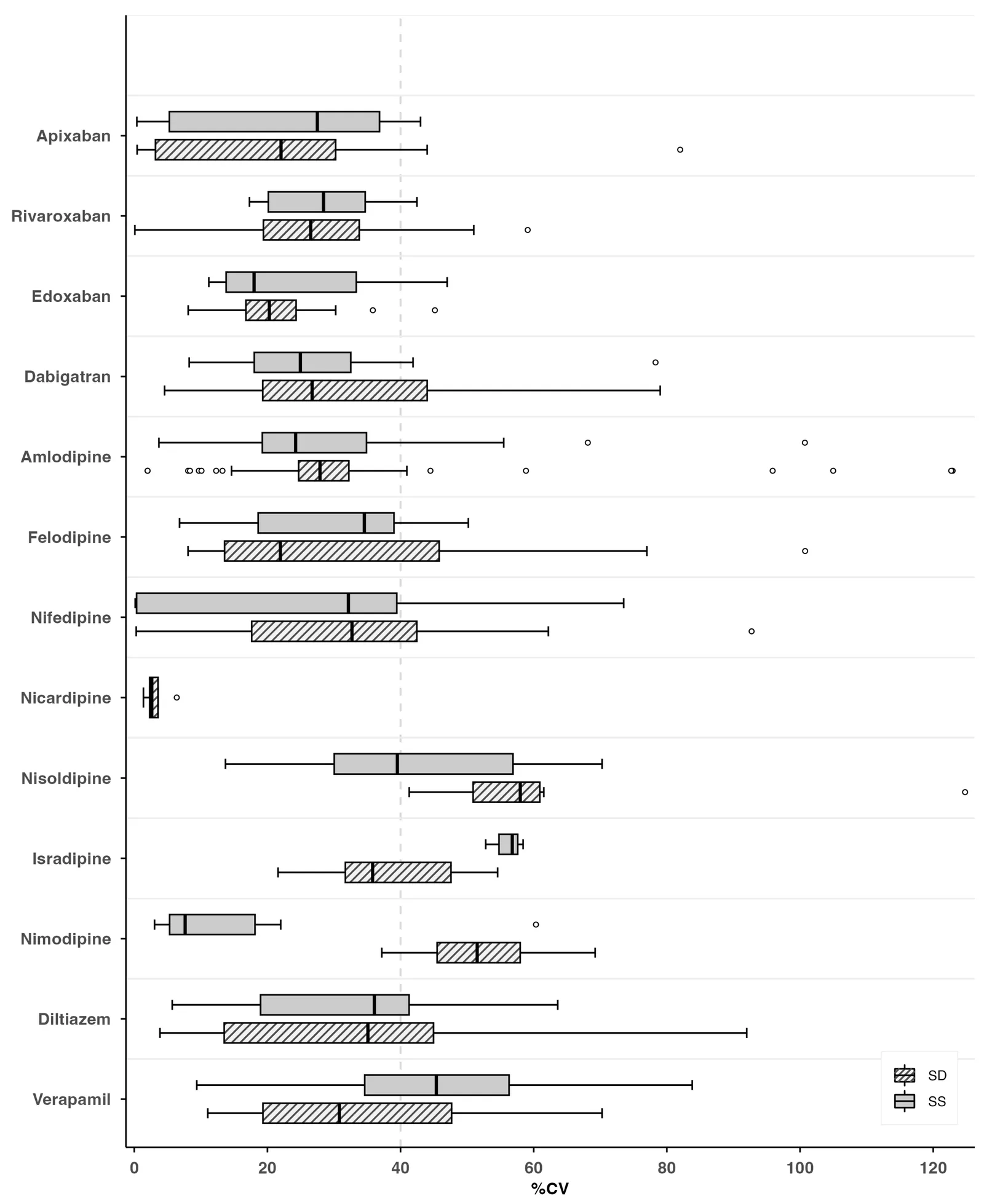
Distribution of the CV% in direct oral anticoagulants (apixaban, rivaroxaban, edoxaban, and dabigatran) and calcium channel blockers (amlodipine, felodipine, nifedipine, nicardipine, nisoldipine, isradipine, nimodipine, diltiazem, and verapamil) for the C_max_ in single-dose (SD) studies and steady-state (SS) studies. The dashed line represents a CV of 40%, the threshold for high PK variability.

**Figure 4:**
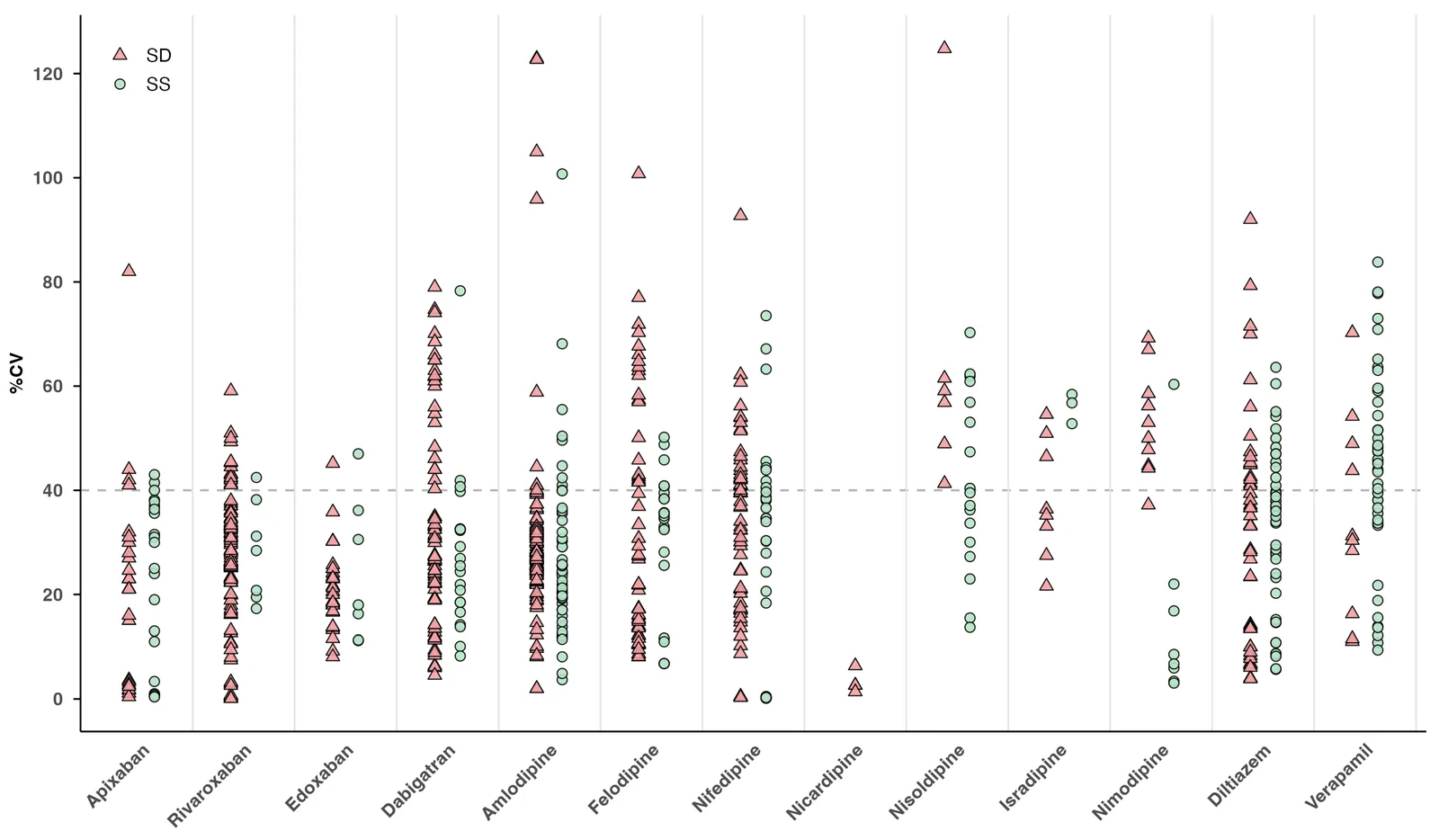
Distribution of the coefficient of variance (CV) in direct oral anticoagulants (apixaban, rivaroxaban, edoxaban, and dabigatran) and calcium channel blockers (amlodipine, felodipine, nifedipine, nicardipine, nisoldipine, isradipine, nimodipine, diltiazem, and verapamil) for the area under the plasma-concentration time curve (AUC) in single-dose (SD) studies and steady-state (SS) studies. The dashed line represents a CV of 40%, the threshold for high PK variability.

Figure 3 and Figure 4 provide a graphical representation of the distribution of CV values for peak and total systemic exposure, respectively. Overall, DOACs exhibited narrower variability distributions than CCBs. As previously documented, dabigatran shows the broadest distribution of CV values, while edoxaban demonstrates the narrowest. Within the CCB class, amlodipine and felodipine generally present lower variability, albeit with notable outliers. The case of amlodipine is particularly interesting, as most studies report low variability; however, several outliers exceeding 80–100% CV suggest that specific populations or experimental conditions may substantially increase its PK variability. Further, these findings support our previous thoughts: nisoldipine, isradipine, nimodipine, diltiazem, and verapamil consistently emerge as compounds requiring careful clinical consideration, displaying broader distributions and a greater number of extreme observations.

### 3.2. Statistical Analysis of PK Variability

To investigate whether PK variability differed between individual agents, CV% values were compared within each drug class, separately for C_max_ and AUC and according to study design (SD and SS). Descriptive statistics for CV values across compounds, PK parameters, and study design are provided in Supplementary Materials (Table S1).

For C_max_, no statistically significant differences in CV distributions were observed among DOACs in either SD (χ^2^ = 5.693, df = 3, *p* = 0.128) or SS studies (χ^2^ = 2.215, df = 3, *p* = 0.529). Although our previous descriptive analysis suggested some variation in CV% across individual DOACs, these were not statistically significant. For AUC, an overall difference was detected in SD studies (χ^2^ = 9.288, df = 3, *p* = 0.026), whereas no difference was observed under SS conditions (χ^2^ = 0.768, df = 3, *p* = 0.857). Notably, despite the significant omnibus result observed for SD studies, none of the individual pairwise comparisons showed significance following post-hoc testing.

A different pattern was identified for CCBs. Significant between-drug differences in C_max_ variability were identified in both SD (χ^2^ = 62.330, df = 8, *p* < 0.001) and SS studies (χ^2^ = 66.393, df = 8, *p* < 0.001). For AUC, significant differences were likewise observed in both SD (χ^2^ = 42.825, df = 8, *p* < 0.001) and SS studies (χ^2^ = 38.449, df = 7, *p* < 0.001). The complete set of pairwise comparisons is presented in Supplementary Materials (Table S2).

### 3.3. Population-Related Sources of PK Variability

**Figure 5:**
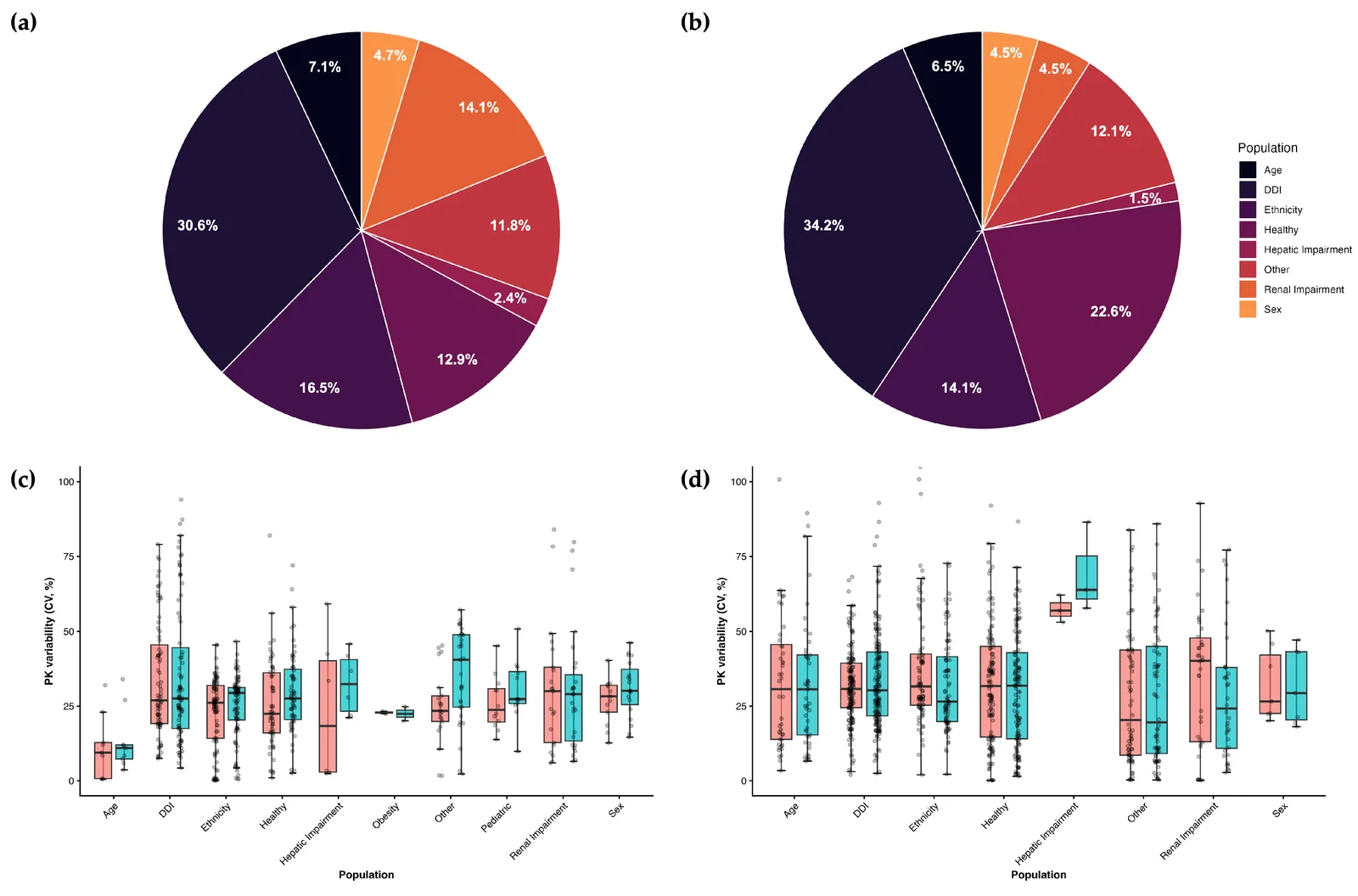
Population sources of PK variability and their impact on drug exposure variability among DOACs and CCBs: (**a**) distribution of the study populations included in PK studies of DOACs, categorized as healthy volunteers, elderly individuals, pediatric populations, obesity, renal impairment, hepatic impairment, sex-specific populations, and DDI studies; (**b**) distribution of study populations included in PK studies of CCBs according to the same population categories; (**c**) distribution of PK variability, expressed as CV (%) across population subgroups for DOACs; and (**d**) distribution of PK variability (CV, %) across population subgroups for CCBs. In panels (**c**,**d**), pink represents C_max_ and blue represents AUC. Boxes represent the interquartile range, horizontal lines indicate the median, whiskers represent the range of values within 1.5× the interquartile range, and individual points represent extracted CV values.

In addition, we characterized our study collection (Figure 5) in terms of the different populations included and/or clinical scenarios investigated. Studies were categorized according to their primary focus, including DDIs, ethnicity, renal impairment, hepatic impairment, age, sex, and other conditions such as obesity, food and herbal interactions, altered physiological states (e.g., gastric transit time), and pregnancy. Studies classified as “healthy” refer to individuals without comorbidities beyond the clinical condition for which the DOAC/CCB is prescribed, or to subjects without any disease or special characteristic that would place them in other predefined categories. DDI studies represented a large proportion of the available literature for both drug classes. Indeed, our DOAC dataset predominantly included studies involving different ethnic groups, individuals with renal impairment, and healthy populations. Similarly, CCB studies were mainly conducted in healthy subjects, across different ethnicities, and in patients with renal impairment.

Substantial heterogeneity in PK variability is recognized for both DOACs and CCBs (Figure 5c,d). In the DOAC dataset, elderly populations showed comparatively lower and more concentrated CV distributions for both C_max_ and AUC, whereas DDI, hepatic impairment, and renal impairment studies displayed broader distributions. A similar degree of heterogeneity was observed across the population categories represented in the CCB PK library, particularly for DDI, ethnicity, hepatic impairment, and renal impairment.

The distribution of PK variability was subsequently compared statistically across the different population categories represented in the included studies. For DOACs, a significant difference in CV distributions was observed across population categories (χ^2^ = 22.746, df = 9, *p* = 0.0068). Post-hoc pairwise comparisons (Supplementary Materials Table S3) identified differences between studies involving elderly and other populations for C_max_, as well as between DDI and elderly populations for AUC. Among CCB studies, the results also indicated statistically significant differences (χ^2^ = 17.117, df = 7, *p* = 0.0167). However, no specific population pair could be identified as significantly different. These findings indicate that the descriptive patterns of PK variability differed across population categories and were not necessarily consistent between drug classes. Notably, renal or hepatic impairment, characterized by marked dispersion in the descriptive analysis, did not translate into statistically significant differences, highlighting the potential influence of drug- and study-specific characteristics.

### 3.4. Multivariate Analysis of PK Variability

**Figure 6:**
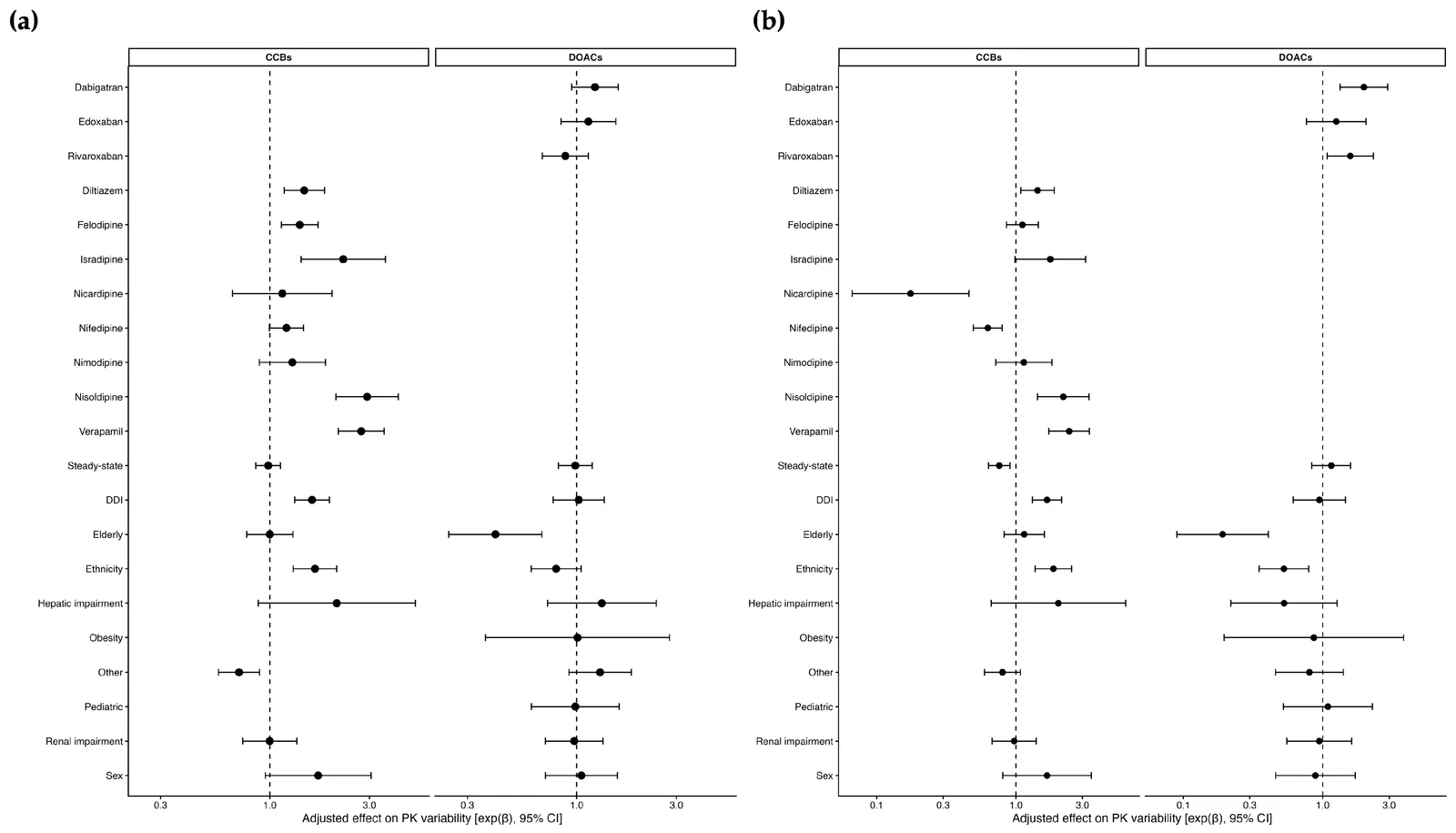
Adjusted associations between drug, study design, population, and PK variability. Estimates represent the adjusted associations between individual drugs, study design, and study population and the CV for (**a**) C_max_ and (**b**) AUC. Estimates were obtained from multivariable linear regression models after log-transformation of CV values and are presented as exponentiated regression [exp(β)] with 95% confidence intervals. Models were fitted separately for DOACs and CCBs and adjusted for drug, study design, and study population. The dashed vertical line at 1 indicates no association. Reference categories were apixaban for DOACs, amlodipine for CCBs, SD studies for study design, and healthy for study population.

Finally, multivariable linear regression models were developed to characterize factors associated with PK variability. Within each drug class, the models included individual drug, study design (SD vs. SS), and study population as explanatory variables, allowing the association between each factor and PK variability to be evaluated while adjusting for the other covariates. The adjusted estimates and corresponding 95% confidence intervals are presented in Figure 6, with complete regression results provided in Supplementary Table S4.

Overall, there was no evidence of significant differences in C_max_ variability among the DOACs after adjustment for study design and population (*p* > 0.05). Although dabigatran had previously shown descriptively higher variability, this difference was not independently associated with drug identity in the multivariable analysis. Only studies involving elderly populations differed significantly from those involving healthy populations (β = −0.896, *p* < 0.001), corresponding to an approximately 59% lower CV. No significant associations were observed for the other population subgroups, including renal impairment (*p* = 0.866). Thus, some of the patterns observed in the exploratory descriptive analysis were not supported after multivariable adjustment. Regarding systemic exposure variability, dabigatran was significantly associated with higher AUC variability (β = 0.680, *p* < 0.001), corresponding to an approximately 1.97-fold higher CV compared with apixaban, as was rivaroxaban (β = 0.455, *p* = 0.020), corresponding to an approximately 1.58-fold higher CV. Ethnicity-specific studies were also associated with lower AUC variability (47% lower CV; β = −0.643, *p* = 0.002).

For CCBs, the multivariable model for C_max_ variability had an adjusted R^2^ of 0.176, indicating that drug identity, study design, and study population collectively accounted for 17.6% of the observed variability in CV. Several CCBs were independently associated with higher C_max_ variability compared with amlodipine, including diltiazem (β = 0.380, *p* < 0.001), felodipine (β = 0.329, *p* = 0.001), isradipine (β = 0.809, *p* < 0.001), nisoldipine (β = 1.072, *p* < 0.001), and verapamil (β = 1.007, *p* < 0.001). In particular, the estimated CV was approximately 2.25-fold higher for isradipine, 2.92-fold higher for nisoldipine, and 2-74-fold higher for verapamil. Nifedipine showed a borderline association with higher variability (β = 0.183, *p* = 0.057), but did not reach the predefined significance threshold. Studies involving DDI populations showed 59% higher CVs than studies involving healthy individuals (β = 0.466, *p* < 0.001), while ethnicity-specific studies showed approximately 64% higher CVs (β = 0.497, *p* < 0.001). In turn, AUC variability was also significantly associated with drug identity: (a) diltiazem (43% higher AUC variability; β = 0.357, *p* = 0.011), (b) nisoldipine (2.19-fold higher CV; β = 0.782, *p* < 0.001), (c) verapamil (2.41-fold higher CV; β = 0.879, *p* < 0.001); (d) nicardipine (82% lower CV; β = −1.741, *p* < 0.001), and (e) nifedipine (37% lower CV; β = −0.466, *p* < 0.001). Unlike the DOAC models, study design was significantly associated with AUC variability among CCBs, with SS studies showing approximately 24% lower CVs than SD studies (β = −0.276, *p* = 0.002). Regarding study population, DDI studies were associated with 67% higher CVs (β = 0.513, *p* < 0.001), whereas ethnicity-specific populations showed 86% higher CVs (β = 0.618, *p* < 0.001) compared with healthy populations.

Although all four models were statistically significant, their explanatory power was modest, with adjusted R2 values ranging from 6.7% to 17.6%. This indicates that drug identity, study design, and study population accounted for only a limited proportion of the observed heterogeneity in PK variability, suggesting that additional sources of variability not captured by the available study-level characteristics may contribute to the observed CVs.

## 4. Discussion

Our study revealed heterogeneity in reported interindividual PK variability across DOACs and CCBs, with important differences in the patterns observed for peak and total systemic exposure. In particular, dabigatran, nisoldipine, isradipine, and nimodipine exhibited a high frequency of studies reporting elevated PK variability (CV > 40%), together with diltiazem and verapamil, which can be described as highly heterogeneous drugs. However, these descriptive differences were not supported by our statistical analysis. For DOACs, no significant differences in C_max_ variability were identified, while dabigatran and rivaroxaban were independently associated with higher AUC variability. In contrast, significant differences between CCBs were observed for both PK metrics, with nisoldipine and verapamil associated with higher variability.

### 4.1. Does Pharmacokinetic Variability in DOAC Therapy Matter?

DOACs are currently recommended and will likely remain the first-line anticoagulant therapy, demonstrating good efficacy in reducing the risk of VTE and stroke in patients with AF [5]. Nevertheless, variability in therapeutic response may lead to ADRs associated with both thromboembolic and bleeding events [300]. Consequently, understanding the relationship between dose, plasma drug concentrations, and successful or unsuccessful therapeutic outcomes is essential for treatment optimization. Evidence indicates that low trough and SS trough concentrations of dabigatran and edoxaban are associated with an increased risk of ischemic stroke [5]. Furthermore, findings from RE-LY and ENGAGE AF-TIMI 48 trials demonstrated that the probability of major bleeding events increases with rising trough plasma concentrations of DOACs [15,301,302]. Although the FDA does not define standardized plasma concentration thresholds for DOAC toxicity, concentrations exceeding 400 ng/mL may be considered indicative of markedly elevated exposure, as this value is substantially higher than the peak concentrations generally expected following conventional therapeutic dosing. Nevertheless, no universal plasma concentration threshold for DOAC toxicity has been established, and the clinical interpretation of drug concentrations remains challenging. This is particularly relevant given the substantial interindividual PK variability observed for some DOACs in the present study, which may result in considerable differences in systemic exposure among patients receiving the same dose. Therefore, our study highlights the clinical importance of PK variability and supports the need to identify patient-specific factors that influence DOAC exposure.

Our analysis did not demonstrate significant differences among the four evaluated DOACs. The descriptive analysis suggested that apixaban, rivaroxaban, and edoxaban exhibited relatively stable PK profiles, with only a limited number of studies reporting C_max_ and AUC CV above 40%, suggesting these agents may be less susceptible to PK variations arising from external factors. Not surprisingly, dabigatran revealed a large proportion of studies reporting CV values above 60%. However, these descriptive patterns were not supported by our statistical analysis. No significant differences in PK variability were identified between DOACs in either SD or SS studies. Nevertheless, after adjustment for study design and population, dabigatran and rivaroxaban were independently associated with higher AUC variability than apixaban. These findings are consistent with previous reports describing IIV in dabigatran exposure and therapeutic response, even under the controlled setting of clinical trials [300].

The variability observed in systemic exposure poses important implications for clinical decision-making. The selection of the most appropriate anticoagulant should consider patient-specific characteristics, particularly renal function and the potential for DDIs, given that these agents undergo varying degrees of renal excretion and are substrates of cytochrome P450 (CYP) enzymes and/or transport proteins implicated in numerous DDIs [300]. Current regulatory recommendations also support therapeutic monitoring and dose adjustment in situations such as renal or hepatic impairment, suspected overdose, before invasive surgical procedures, in cases of non-adherence, following bleeding or thrombotic events, or whenever altered drug disposition is suspected [303].

### 4.2. Key Covariates Influencing DOAC Exposure: Age and Ethnicity

Within our study library, age emerged as an important population characteristic associated with PK variability. Studies involving elderly populations showed a 59% lower CV for C_max_ and 81% lower CV for AUC compared with studies conducted in healthy populations. However, these findings should not be interpreted as evidence that DOAC exposure is more predictable in older patients. Rather, the observed associations may reflect characteristics of the available evidence, including study conditions, drug composition, and the distribution of clinical characteristics within the elderly populations represented in the literature. A RE-LY analysis identified age as the most important covariate affecting dabigatran disposition, with patients older than 75 years exhibiting a 68% increase in serum concentrations compared with those younger than 65 years [302]. Although we did not evaluate the effect of individual covariates separately for each drug, our findings are broadly consistent with previous evidence reporting that apixaban is the DOAC with the most stable therapeutic levels, compared with rivaroxaban and dabigatran, which have presented supratherapeutic concentrations in elderly patients [304,305]. Despite these concerns, the benefits of anticoagulation generally outweigh the risk of ADRs in the older population [306]. Nevertheless, implementation of DOAC monitoring in elderly patients, particularly those with impaired renal function and multiple comorbidities associated with a higher risk of DDIs, may be clinically beneficial [5].

Polypharmacy is particularly relevant in the context of DOAC therapy. In fact, high PK variability was most frequently observed in studies evaluating DDIs in dabigatran-treated patients (Supplementary Materials Figure S1). Similar patterns were identified for apixaban and rivaroxaban, whereas no edoxaban study reported CV values above 40%. The PK properties of DOACs provide a mechanistic explanation for these findings. Rivaroxaban and apixaban are substrates of CYP3A4/5 and CYP2J2, and CYP3A4/5, respectively, and efflux transporters such as P-glycoprotein (P-gp) and breast cancer resistance protein (BCRP) [307,308,309,310,311]. Dabigatran, in contrast, does not undergo CYP-mediated metabolism but rather is metabolized through acylglucuronidation [312]. Furthermore, dabigatran etexilate is a prodrug of dabigatran and a substrate of P-gp, exhibiting an absolute bioavailability of only 6.5%, making it particularly susceptible to transporter-mediated interactions [90,313]. The expression and activity of these enzymes and transporters play a critical role in modulating DOAC disposition [300]. Concomitant medications acting as substrates, inhibitors, or inducers of both P-gp and CYP enzymes can alter DOAC exposure, and this may result in adequate anticoagulation or an increased risk of bleeding. For instance, verapamil, a potent P-gp inhibitor, increases dabigatran AUC by approximately 2.4-fold when administered one hour before dabigatran, and by 71% when coadministered [96]. Indeed, major bleeding events have been reported in elderly patients receiving dabigatran concomitantly with P-gp inhibitors, potentially as a consequence of P-gp inhibition combined with advanced age and impaired renal function [314,315,316].

In contrast, because edoxaban undergoes limited metabolism via CYP3A4 [317], clinically relevant interactions with CYP inducers or inhibitors are generally less pronounced. Consequently, concomitant administration of medications such as cyclosporine, dronedarone, and ketoconazole is strongly discouraged with dabigatran and rivaroxaban [15,318]. As argued by Gong et al. [300], it remains unclear whether the cumulative effect of multiple moderate inhibitors on DOAC PK-PD is equivalent to that of a single potent inhibitor. This represents an important gray area in clinical practice, as the full spectrum of these interactions has yet to be comprehensively characterized in real-world populations. Until then, dose adjustments (Table 6), together with appropriate therapeutic monitoring, should be implemented whenever clinically indicated. Importantly, despite the established pharmacological relevance of DDIs, this was not identified as an independent predictor of PK variability in our study.

Ethnicity was another population characteristic significantly associated with AUC variability, demonstrating an approximately 47% lower CV compared with healthy populations. This finding should not be interpreted as evidence that ethnicity itself reduces DOAC PK variability. Rather, differences in genetic background, environmental factors, dietary habits, body composition, and other population characteristics may contribute to differences in drug disposition between populations. Ethnicity is closely related to genetic polymorphism affecting the expression of genes involved in ADME and may therefore contribute to interindividual and interpopulation differences in drug exposure [5]. Nevertheless, the available evidence remains insufficient to support routine DOAC dose adjustment based solely on ethnicity.

Although renal impairment was not associated with PK variability in our regression model, the descriptive analysis shows that renal function is not irrelevant to DOAC exposure. Current guidelines recommend dose reduction or specific dosing considerations for several DOACs according to renal function (Table 6). The absence of a significant association in our analysis may reflect the complex relationship between kidney function, age, comorbidities, and drug-specific excretion pathways. In fact, approximately 80% of circulating dabigatran is eliminated via the kidneys, whereas only 33% and 25% of rivaroxaban and apixaban, respectively, are renally cleared [307,319,320]. Consequently, dabigatran is the DOAC most susceptible to variability driven by renal function. Chronic kidney disease (CKD) is not uncommon in patients with AF; thus, the use of DOACs in this population requires careful consideration due to altered drug clearance and the potential for increased risk of bleeding and stroke [6,321]. Although dosing recommendations exist for CKD patients, evidence in those with advanced CKD (CrCl < 15 mL/min) remains limited, as these patients are generally excluded from phase III clinical trials. In this population, apixaban is often preferred due to its lower degree of renal excretion [6].

Current guidelines recommend that DOACs should be used with caution in patients with severe renal impairment when CrCl is 15–29 mL/min, except for dabigatran, which is contraindicated in this range [6,322]. In patients with mild and moderate renal impairment, reduced DOAC doses must be considered (Table 6). In addition, edoxaban is not recommended in patients with CrCl > 95 mL/min due to reduced efficacy in this subgroup [322]. PK modeling is once again valuable for predicting drug efficacy and safety. For example, a study of rivaroxaban suggested that CrCl-based dosing may be more accurate than standard dosing, proposing a modification from once-daily to twice-daily dosing in patients with CrCl ≥ 70–159 mL/min (10 mg) and in patients with CrCl ≥ 160 mL/min (15 mg) [323].

One study from our library in Caucasian patients with moderate renal impairment receiving dabigatran etexilate 150 mg once daily reported a CV of 79.8% for C_max_ and 78.3% for AUC [114]. These findings indicate that even in individuals with moderate renal dysfunction, systemic exposure varies. This emphasizes the importance of considering the full patient profile rather than focusing on a single characteristic. Indeed, patients with CKD are often older, and aging itself is associated with an increased risk of DOAC accumulation. The RE-LY analysis demonstrated a strong correlation between age and reduced renal function [302,324,325]. Further, older adults present a higher prevalence of chronic conditions and are more likely to be exposed to polypharmacy, which predisposes them to DDIs [324,325]. Although our analysis identified elderly populations as being associated with lower reported PK variability, this result should not be interpreted as evidence that aging protects against excessive drug exposure or toxicity. On the contrary, advanced age is a well-established risk factor for altered drug disposition and ADRs. The apparent discrepancy between lower CV values in elderly populations and the established clinical vulnerability of older patients further illustrates the distinction between variability in aggregated PK estimates and the clinical risk associated with individual drug exposure.

Additional patient-specific factors may also influence the PK profile of DOACs with clinically relevant consequences. At present, dose adjustment based on BMI is not recommended, although rivaroxaban and apixaban are often preferred in obese patients [326]. For other covariates, there is currently insufficient evidence to support routine dose adjustments.

### 4.3. Pharmacokinetic Variability Among CCBs: Clinical Implications

Approximately 30–50% of patients receiving CCB therapy for hypertension management fail to achieve their target blood pressure (BP) goals [327,328,329,330]. Variations in CCB serum concentrations resulting from interindividual PK variability represent one of the factors contributing to uncontrolled hypertension [330]. Furthermore, when plasma concentrations exceed the toxicity threshold reported for a given CCB, adverse effects may occur, including suppression of sinoatrial (SA) and atrioventricular (AV) node activity, leading to bradycardia and subsequent hypotension. Episodes of excessive vasodilation may also occur, particularly with DHPs [19,331,332]. These vasodilatory effects can result in headaches, flushing, hypotension, and peripheral edema [333,334]. Regulatory agencies have established no standardized plasma concentration thresholds for CCB toxicity. For diltiazem, therapeutic blood concentrations have been reported to range from 20–200 ng/mL, whereas therapeutic concentrations of verapamil are generally reported between 80–400 ng/mL; concentrations exceeding these ranges may indicate increased systemic exposure and a greater potential for toxicity. However, these values should not be interpreted as definitive toxicity thresholds. The toxic dose of diltiazem in humans remains unknown, and its extensive metabolism may result in more than tenfold variation in blood concentrations following standard dosing, limiting the clinical utility of plasma concentrations for predicting toxicity. Similarly, the safety and clinical relevance of verapamil concentrations above the conventional therapeutic range remain incompletely characterized.

Therefore, identifying the factors that influence the PK profile of CCBs may be important for optimizing antihypertensive therapy. The qualitative evidence summarized in Table 1 suggested that several CCBs are associated with wide interindividual PK variability. Our work extends this evidence by quantitatively characterizing PK variability across a broad range of studies and patient populations. We found that, among all CCBs, nisoldipine, isradipine, and nimodipine demonstrated the highest proportion of studies reporting high PK variability, while diltiazem and verapamil were characterized by marked heterogeneity in their PK profiles. The multivariable analysis further refined these descriptive findings, demonstrating that several compounds remained independently associated with higher variability after adjustment for study design and population. The most relevant associations were observed for nisoldipine and verapamil, which showed higher variability for both C_max_ and AUC compared with amlodipine. From a clinical perspective, as systemic exposure to these agents varies considerably among patients receiving the same dose, treatment response may become less predictable, potentially resulting in either suboptimal BP control or an increased risk of ADRs. This may be particularly relevant for verapamil, given its negative chronotropic and ionotropic effects. Excessive systemic exposure may increase the risk of bradycardia and worsen cardiac output [332].

Contrary to our findings, Rognstad et al. [330] investigated the PK variability of amlodipine and its effects on blood pressure control in patients treated for hypertension. Their study concluded that female sex, advanced age, reduced renal function, and decreased CYP3A4 enzymatic activity were associated with higher dose-adjusted serum concentrations of amlodipine. Furthermore, other studies have underscored that hepatic impairment reduces amlodipine clearance, resulting in a 40–60% increase in systemic exposure and, consequently, the need for dose adjustment [330]. In contrast, renal function appears to have only a minor effect on amlodipine PK, and dose adjustment is generally not considered necessary in patients with renal impairment [335,336]. Taken together, the available evidence remains somewhat controversial. Indeed, our findings point toward low PK variability for amlodipine, suggesting that the impact of patient-specific factors on its disposition may be less pronounced than previously reported. Further investigations are warranted to better characterize the sources and extent of PK variability associated with amlodipine therapy.

### 4.4. Are Some Patients Predisposed to Altered CCB Exposure?

According to current guidelines, CCB dosing varies depending on the specific compound and patient characteristics. For example, the recommended initial dose of amlodipine for uncomplicated hypertension is typically 5 mg once daily and may be increased to 10 mg once daily according to the individual patient’s response [337]. As noted above, our findings suggest that amlodipine is not a particularly concerning compound with respect to PK variability. In contrast, other CCBs appear to be more susceptible to variability. Nifedipine treatment is generally initiated at 30 mg once daily and may be titrated up to 60–90 mg once daily, particularly in specific populations such as elderly patients or those with impaired renal function [338].

DDI status emerged as an independent predictor of PK variability, being associated with approximately 59% higher C_max_ variability and 67% higher AUC variability compared with healthy populations. These findings support the relevance of concomitant medication use as an important source of variability in systemic CCB exposure. Individual studies within our study library further illustrate the potential clinical consequences of such interactions. One example is the interaction between nifedipine and apatinib, a vascular endothelial growth factor receptor-2 (VEGFR-2) inhibitor indicated for advanced gastric adenocarcinoma and gastroesophageal junction cancer [217]. This interaction is clinically relevant because hypertension is a common adverse effect of vascular-targeting anticancer agents such as apatinib, making nifedipine a potential co-medication in routine clinical practice. To investigate this, Zhu et al. [217] conducted a single-center, open-label, single-arm clinical study in patients with advanced solid tumors. Participants received a single 30 mg dose of nifedipine followed by daily administration of 750 mg apatinib. The authors demonstrated that coadministration increased nifedipine systemic exposure by 83%, confirming a clinically relevant PK interaction mediated by CYP3A4 inhibition. Interestingly, the reported CV values for both C_max_ and AUC were approximately 40%, suggesting a moderate variability within the study population. These findings illustrate an important concept: even when a DDI alters the PK profile, the magnitude of its clinical effects may differ considerably between individuals. In other words, some patients may experience clinically relevant exposure increases, while others may not, depending on additional patient-specific factors that further contribute to variability. This reinforces the notion that clinicians should not evaluate individual patient characteristics in isolation but rather consider the complete patient profile when selecting and adjusting therapeutic regimens. In the study conducted by Zhu et al. [217], it would be particularly informative to identify the individuals who experienced the greatest increases in nifedipine exposure, rather than relying exclusively on population-average estimates. Such an approach could help elucidate the physiological and clinical factors that may exacerbate the magnitude of the DDI in specific patients. This would be valuable for implementing precision dosing strategies, allowing apatinib-nifedipine co-therapy to be tailored according to an individual’s risk of nifedipine toxicity.

Indeed, CCBs constitute a pharmacologically diverse drug class that is frequently administered in combination with other therapeutic agents, as hypertension commonly coexists with multiple chronic conditions [339]. Thus, polypharmacy is highly prevalent in this population. Nimodipine, one of the compounds identified in our analysis as exhibiting high IIV, was evaluated in three DDI studies out of nine included in our study library. Similarly, three of the seven studies available for nisoldipine investigated DDI scenarios. The highest mean PK variability for both compounds was observed in DDI settings, suggesting that concomitant medication use represents a major contributor to variability in systemic exposure for these DHPs. The PK characteristics of nisoldipine provide a plausible mechanistic explanation for these findings. As a substrate of CYP3A4, nisoldipine is susceptible to alterations in exposure following coadministration with CYP3A4 inhibitors or inducers. Such interactions may result in reduced plasma concentration and loss of antihypertensive efficacy or, conversely, excessive systemic exposure and an increased risk of dose-dependent adverse effects [332]. In addition to DDIs, age emerged as a relevant source of variability in our analysis. This observation is consistent with current prescribing recommendations, which advocate a lower initial dose of 8.5 mg once daily in elderly patients due to the potential for increased plasma drug concentrations [340]. A similar pattern was observed for diltiazem, for which the highest mean variability was identified in studies involving DDIs and elderly populations. These findings suggest that advanced age and concomitant medication use may constitute particularly important determinants of PK variability in patients receiving diltiazem or nisoldipine. Supporting this hypothesis, Bianchetti et al. [281], in a study included in our database, demonstrated that elderly individuals exhibit increased diltiazem bioavailability due to reduced first-pass metabolism.

Ethnicity was also a population characteristic associated with PK variability. In the multivariable analysis, ethnicity-specific populations were associated with approximately 64% higher C_max_ variability and 86% higher AUC variability compared with healthy populations. This suggests that differences between populations may contribute to the heterogeneity observed in CCB exposure. Again, this association should not be interpreted as evidence that ethnicity itself is a direct determinant of PK variability. Further investigation is required to establish whether specific genetic or physiological factors underlie the observed association.

Renal function also represents a clinically relevant consideration in CCB therapy, particularly because hypertension affects approximately 80% of patients with CKD [341], making the optimization of antihypertensive therapy in this population a big challenge. The available literature generally documents that the PK parameters of most CCBs are comparable between healthy individuals, patients with CKD, and those with end-stage renal disease [342]. Our findings support this. For instance, Harten et al. [235] reported no significant differences in mean nisoldipine PK parameters between subjects with renal impairment and healthy controls. Nevertheless, all reported CV values exceeded 40%, indicating clear IIV in drug disposition despite the absence of statistically significant differences in average exposure. These findings highlight the limitations of relying solely on mean PK estimates when evaluating drug kinetics in special populations.

Additional evidence of clinically relevant variability was observed for verapamil. Several studies included in our library reported highly variable C_max_ and AUC values under specific conditions, including food interactions (Supplementary Materials Figure S1). In a study conducted by Ho et al. [296], administration of grapefruit juice twice daily in combination with verapamil 120 mg twice daily resulted in a significant increase in systemic exposure. This effect was attributed to the inhibition of intestinal CYP3A4, leading to reduced presystemic metabolism and enhanced oral bioavailability. Given that grapefruit consumption should be discouraged in CCB-treated patients, as excessive exposure may increase the risk of toxicity [342].

Based on our findings, we identified clinical scenarios and patient populations in which CCB dose individualization may warrant further investigation (Table 7). However, these endorsements should be interpreted with caution, as they are derived from variability estimates reported in the literature. Prospective clinical and pharmacometrics studies are required to establish the flag covariates and to support evidence-based personalized dosing strategies for CCB therapy.

### 4.5. Future Directions in Exposure-Guided and Personalized DOAC and CCB Therapy

The assumption that PK variability is uniformly low or clinically negligible in DOACs and CCBs is therefore challenged by our findings. Based on this, routine therapeutic monitoring of DOACs and CCBs may be valuable in clinical scenarios characterized by unexpectedly low or undetectable serum concentrations, as well as in cases of excessively high exposure. Moreover, the identification of key patient characteristics that should be considered when prescribing these dosing regimens highlights specific subgroups that may benefit from TDM. This may help reduce clinical inertia in dose adjustment decisions. Real-world evidence indicates that many patients receive doses that are inconsistent with labeled recommendations, including under- and overdosing. For instance, clinicians often fear the risk of bleeding, particularly in elderly patients with AF, leading to frequent underdosing. Conversely, overdosing may occur when renal function is not adequately assessed at the time of prescription or when patient-specific characteristics change during treatment. TDM may help prevent such situations [330]. In particular, direct measurement of plasma drug concentrations represents a potentially optimal and accurate approach for monitoring exposure; however, we are aware of the limitations associated with this strategy, including feasibility and accessibility in routine clinical practice.

Beyond conventional TDM, virtual and digital twin technologies represent promising future approaches to support precision dosing. Although these technologies are not yet part of routine clinical practice, they may eventually enable individualized prediction of drug exposure and therapeutic response through integration of patient-specific physiological and clinical data. Their potential role should therefore be viewed as a future research direction. A virtual twin (VT) generally consists of a patient-specific computational model that incorporates demographic, physiological, genetic, and clinical information to predict individual drug exposure and response. However, whenever new patient information becomes available, the model requires re-simulation to update its predictions. In contrast, a digital twin (DT) represents a more advanced and integrated framework in which the virtual representation of the patient is continuously synchronized with real-world data through automated data streams. As a result, DTs can dynamically update model states over time, providing a holistic representation of the individual and enabling real-time prediction [343].

The integration of TDM data with VT and DT technologies may represent a major step toward precision dosing of DOACs and CCBs. By combining measured drug concentrations with patient-specific covariates, these platforms may improve the prediction of individual PK profiles and support proactive dose optimization. Such approaches could be particularly valuable for identifying patients at risk of subtherapeutic or supratherapeutic exposure.

### 4.6. Limitations

Several limitations should be considered when interpreting these findings. First, the number of available studies differed considerably across compounds. While amlodipine, rivaroxaban, and dabigatran were represented by extensive datasets, other drugs such as nicardipine, isradipine, and nisoldipine were evaluated in a limited number of studies. Therefore, the variability profiles of some therapeutic agents should be interpreted cautiously, as a small number of studies may not fully capture the true extent of PK variability observed in clinical practice. Second, the included studies encompassed diverse populations, study designs, analytical methodologies, dosing regimens, formulations, and sampling strategies. The study populations differed in terms of age, sex, ethnicity, disease status, renal and hepatic function, concomitant medications, and other patient-specific characteristics. Although this heterogeneity reflects the complexity of real-world populations and was a deliberate feature of our study design, it may also contribute to differences in reported CV values that are not exclusively attributable to biological interindividual variability. In particular, differences in study design and bioanalytical methodology (analytical platforms, assay procedures, and associated performance characteristics) may introduce additional sources of variability that cannot be disentangled from patient-related factors using aggregated literature data. Nevertheless, this broad population spectrum was considered essential for the primary objective of the present work, which was to characterize the range of PK variability reported across clinically relevant scenarios rather than within a single homogeneous population. Importantly, conventional clinical trials frequently rely on highly selected and relatively homogeneous populations, which may not adequately represent the diversity encountered in routine clinical practice. Therefore, although population heterogeneity limits direct comparisons between studies, it also provides valuable insight into how differences in patient characteristics and clinical conditions may contribute to variability in drug exposure. Future analyses based on individual patient-level data will be necessary to disentangle the independent contribution of these factors and quantify their respective effects on PK variability. Third, PK variability was assessed using only two exposure metrics (C_max_ and AUC) and, despite being widely accepted indicators of peak and overall systemic exposure, they may not fully capture clinically relevant variations in drug disposition. For DOACs in particular, trough concentrations are often more closely associated with efficacy and safety outcomes. Fourth, the inclusion of multiple PK observations from the same publication, even when they represented distinct experimental conditions, may have increased the representation of compounds that have been more extensively studied. This approach was intentionally adopted to capture the full spectrum of PK variability across different clinical scenarios. Nevertheless, this should be considered when interpreting comparisons between compounds. Another limitation is that the classification of PK variability into predefined CV categories and the adoption of a 40% threshold to define high variability should be regarded as a pragmatic rather than a universally accepted approach. Although this threshold has been used in previous PK investigations, the clinical significance of a given CV value may differ according to the therapeutic index, PD properties, and exposure-response relationship of each drug. This should not be interpreted as a universal clinical threshold for clinical decision-making and dose adjustment. Furthermore, this analysis was restricted to studies indexed in PubMed, as relevant studies available in other databases, gray literature, regulatory reports, conference proceedings, or unpublished datasets may not have been captured. Thus, publication bias cannot be excluded. Finally, the present study was designed to characterize and compare PK variability rather than establish causal relationships between specific covariates and clinical outcomes. Consequently, although associations between variability and factors such as DDIs, age, or ethnicity were identified, the magnitude of their independent contribution to variability could not be quantified. Future studies integrating individual patient data, population pharmacokinetic modeling, pharmagenomic information, and real-world TDM datasets are needed to better define the determinants of variability and support precision dosing strategies for both DOACs and CCBs.

## 5. Conclusions

This study provides a comparative assessment of interindividual PK variability in two widely used drug classes, DOACs and CCBs, demonstrating that variability in systemic exposure remains a relevant and clinically meaningful phenomenon across both therapeutic areas. Although DOACs are generally associated with stable PK profiles, the descriptive analysis indicates that dabigatran exhibits high variability, while edoxaban shows the most consistent exposure. In parallel, CCBs displayed an even broader variability profile, ranging from stable compounds such as amlodipine and felodipine to highly heterogeneous agents including nisoldipine, isradipine, nimodipine, diltiazem, and verapamil. Importantly, current clinical guidelines are consistent with these findings, reinforcing the need for clinicians to adhere to evidence-based recommendations for clinical decision-making. These descriptive differences, however, were not supported by formal statistical comparisons. After adjustment for study design and population, dabigatran and rivaroxaban were independently associated with higher AUC variability than apixaban, while DDIs, ethnicity, and specific drug-related factors emerged as relevant determinants of PK variability.

Overall, these findings indicate that PK variability is not uniformly distributed across DOACs and CCBs and is influenced by both drug- and population-related factors. Although this work does not support a universal need for dose adjustment or routine TDM, they highlight specific drugs and patient characteristics that may warrant greater attention when altered drug exposure is suspected. Future individual patient-level analyses integrating population PK and real-world TDM data may help translate these observations into more precise and individualized dosing strategies.

## Figures and Tables

**Table 1:** Interindividual variability associated with calcium channel blocker disposition.

CCB	Interindividual Variability	References
Amlodipine	Intermediate	[29]
Felodipine	Wide	[30]
Nifedipine	Low	[31]
Nicardipine	Wide	[32]
Nisoldipine	Wide	[33]
Isradipine	Wide	[34]
Nimodipine	-	
Diltiazem	Wide	[35]
Verapamil	Wide	[36]

CCB, calcium channel blocker.

**Table 2:** Number of CV observations for C_max_ and AUC in SD and SS studies for each DOAC and CCB.

Drug	Observations for C_max_		Observations for AUC		Total Studies
	SD (*n*) *	SS (*n*) *	SD (*n*) *	SS (*n*) *	
DOACs					
Apixaban	48	22	47	20	15
Rivaroxaban	96	23	98	7	25
Edoxaban	29	12	28	7	11
Dabigatran	85	25	81	20	26
CCBs					
Amlodipine	111	56	113	56	65
Felodipine	61	27	57	23	25
Nifedipine	63	35	62	34	27
Nicardipine	4	4	4	0	3
Nisoldipine	7	15	7	15	7
Isradipine	8	3	8	3	4
Nimodipine	10	9	10	7	9
Diltiazem	41	46	43	47	30
Verapamil	15	47	10	42	17

SD, single-dose; SS, steady-state; DOACs, direct oral anticoagulants; CCBs, calcium channel blockers. * Values represent the number of individual PK CV determinations.

**Table 3:** Pharmacokinetic variability in single-dose (SD) and steady-state (SS) studies of 4 direct oral anticoagulants: apiaban, rivaroxaban, edoxaban, and dabigatran.

Drug	Study Type	CV Parameter	Doses (mg)	CV 0–20%	CV 20–40%	CV 40–60%	CV 60–80%	CV > 80%	References
Apixaban	SD	C_max_	0.5–5	10	30	7	1	0	[38,39,40,41,42,43,44,45,46,47,48,49]
		AUC	0.5–5	19	20	6	1	1	[38,39,40,41,42,43,44,45,46,47,48,49]
	SS	C_max_	2.5–25	7	12	3	0	0	[42,50,51,52,53]
		AUC	2.5–25	8	9	2	0	1	[42,50,51,52,53]
Rivaroxaban	SD	C_max_	2.5–80	26	63	7	0	0	[54,55,56,57,58,59,60,61,62,63,64,65,66,67,68,69,70,71,72,73,74]
		AUC	2.5–80	26	58	14	0	0	[54,55,56,57,58,59,60,61,62,63,64,65,66,67,68,69,70,71,72,73,74]
	SS	C_max_	10–20	8	3	12	0	0	[53,75,76,77,78]
		AUC	10–20	2	4	1	0	0	[53,75,78]
Edoxaban	SD	C_max_	10–150	1	22	6	0	0	[79,80,81,82,83,84,85,86]
		AUC	10–150	13	14	1	0	0	[79,80,81,82,83,84,85,86]
	SS	C_max_	60–120	2	10	0	0	0	[80,87,88,89]
		AUC	60–120	4	2	1	0	0	[80,87,88,89]
Dabigatran	SD	C_max_	75–400	23	37	6	13	6	[90,91,92,93,94,95,96,97,98,99,100,101,102,103,104,105,106,107,108]
		AUC	75–400	21	37	9	14	0	[90,91,92,93,94,95,96,97,98,99,100,101,102,103,104,105,106,107,108]
	SS	C_max_	50–400	6	11	5	3	0	[90,109,110,111,112,113,114,115]
		AUC	50–400	7	10	2	1	0	[90,109,111,112,113,114,115]

**Table 4:** Pharmacokinetic variability in single-dose (SD) and steady-state (SS) studies of 10 calcium channel blockers: amlodipine, felodipine, nifedipine, nicardipine, nisoldipine, isradipine, nimodipine, diltiazem, and verapamil.

Drug	Study Type	CV Parameter	Doses (mg)	CV 0–20%	CV 20–40%	CV 40–60%	CV 60–80%	CV > 80%	References
Amlodipine	SD	C_max_	5–60	40	64	7	0	0	[116,117,118,119,120,121,122,123,124,125,126,127,128,129,130,131,132,133,134,135,136,137,138,139,140,141,142,143,144,145,146,147,148,149,150,151,152,153,154,155,156,157,158,159,160,161,162]
		AUC	5–60	14	88	5	0	6	[116,117,118,119,120,121,122,123,124,125,126,127,128,129,130,131,132,133,134,135,136,137,138,139,140,141,142,143,144,145,146,147,148,149,150,151,152,153,154,155,156,157,158,159,160,161,162]
	SS	C_max_	5–15	25	27	4	0	0	[116,117,122,129,135,147,162,163,164,165,166,167,168,169,170,171,172,173,174,175,176,177,178,179,180]
		AUC	5–15	18	28	7	1	2	[116,117,122,129,135,147,162,163,164,165,166,167,168,169,170,171,172,173,174,175,176,177,178,179,180]
Felodipine	SD	C_max_	0.525–10	26	18	13	4	0	[140,162,181,182,183,184,185,186,187,188,189,190,191,192,193,194,195,196,197,198]
		AUC	0.525–10	26	11	9	10	1	[140,162,181,182,183,184,185,186,187,188,189,190,191,192,193,194,195,196,197,198]
	SS	C_max_	2.5–20	6	9	12	0	0	[162,181,183,184,192,196,199,200,201,202,203]
		AUC	2.5–20	6	12	5	0	0	[162,181,183,184,192,199,200,201,202,203]
Nifedipine	SD	C_max_	3.5–60	17	21	19	5	1	[204,205,206,207,208,209,210,211,212,213,214,215,216,217,218,219,220]
		AUC	4.4–60	17	21	21	2	1	[205,206,207,208,209,210,211,212,214,215,216,217,218,219,220,221,222,223]
	SS	C_max_	10–60	11	7	8	9	0	[205,206,215,221,224,225,226,227,228,229,230]
		AUC	10–60	12	14	5	3	0	[205,206,215,221,223,224,225,226,227,228,229,230]
Nicardipine	SD	C_max_	30	4	0	0	0	0	[231,232]
		AUC	30	4	0	0	0	0	[231,232]
	SS	C_max_	10–40	0	0	2	2	0	[233]
		AUC	10–40	0	0	0	0	0	-
Nisoldipine	SD	C_max_	5–10	0	0	3	1	3	[234,235,236]
		AUC	5–10	0	0	4	1	2	[234,235,236]
	SS	C_max_	5–20	2	3	6	2	2	[234,235,237,238,239]
		AUC	5–20	2	5	4	4	0	[234,235,237,238,239,240]
Isradipine	SD	C_max_	2.5–10	3	0	1	3	1	[241,242,243]
		AUC	2.5–10	0	5	3	0	0	[241,242,243]
	SS	C_max_	5	0	0	2	1	0	[241,244]
		AUC	5	0	0	3	0	0	[241,244]
Nimodipine	SD	C_max_	0.5–60	0	1	4	3	2	[245,246,247]
		AUC	0.5–60	0	1	7	2	0	[245,246,247]
	SS	C_max_	1.6–120	8	1	0	0	0	[248,249,250,251,252]
		AUC	1.6–120	6	1	0	1	0	[248,249,251,252,253]
Diltiazem	SD	C_max_	30–360	16	14	11	0	0	[140,254,255,256,257,258,259,260,261,262,263,264,265,266,267,268,269,270]
		AUC	30–360	14	13	11	3	2	[140,254,255,256,257,258,259,260,261,262,263,264,265,266,267,268,269,270,271]
	SS	C_max_	0.5–60	12	25	9	0	0	[254,255,259,262,264,265,266,269,272,273,274,275,276,277,278,279,280,281,282]
		AUC	0.5–60	12	23	10	2	0	[254,255,262,264,265,266,269,271,272,273,274,275,276,277,278,279,280,281,282]
Verapamil	SD	C_max_	80–240	3	7	3	0	2	[283,284,285,286,287,288]
		AUC	80–240	3	3	3	1	0	[283,284,285,286,288,289]
	SS	C_max_	30–360	4	14	23	5	1	[283,286,288,290,291,292,293,294,295,296,297,298]
		AUC	30–360	7	9	18	7	1	[283,286,288,289,292,294,295,296,297,298,299]

**Table 5:** Distribution of CV for AUC in PK studies of DOACs and CCBs where the CVs were >40% (high variability).

Drug	Number (%) of SD Studies with CV > 40% for AUC	Number (%) of SS Studies with CV > 40% for AUC
Apixaban	5/12 (42%)	3/6 (50%)
Rivaroxaban	6/21 (29%)	1/5 (20%)
Edoxaban	1/8 (13%)	0/3 (0%)
Dabigatran	10/19 (53%)	3/9 (33%)
Amlodipine	7/47 (15%)	5/25 (20%)
Felodipine	7/20 (35%)	4/10 (40%)
Nifedipine	11/20 (55%)	6/12 (50%)
Nicardipine	0/2 (0%)	0/1 (0%)
Nisoldipine	3/3 (100%)	4/6 (67%)
Isradipine	1/3 (33%)	2/2 (100%)
Nimodipine	3/3 (100%)	1/6 (17%)
Diltiazem	10/19 (53%)	10/20 (50%)
Verapamil	3/7 (43%)	8/14 (57%)

SD, single-dose; SS, steady-state; AUC, area under the concentration-time curve; DOACs, direct oral anticoagulants; CCBs, calcium channel blockers.

**Table 6:** Recommended DOAC dose adjustments in special populations and clinical scenarios [300,318].

	Dabigatran (mg BID)	Rivaroxaban (mg OD)	Apixaban (mg BID)	Edoxaban (mg OD)
Age				
75–80 years	110	20	5	60
≥80 years	110	20	2.5	60
Body weight				
50–60 kg	150	20	2.5	30
<50 kg	110	20	2.5	30
Renal impairment				
Mild (CrCl 51–80 mL/min)	150	20	5	30
Moderate (CrCl 30–50 mL/min)	150 or 110	15	5	30
Severe (CrCl < 30 mL/min)	NR	15	2.5	NR
Hepatic impairment				
Mild (Child-Pugh A)	150	20	5	-
Moderate (Child-Pugh B)	150	NR	5	-
Severe (Child-Pugh C)	NR	NR	NR	-
Hepatic dysfunction	NR	NR	NR	-
Drug–drug interactions				
P-gp inhibitor	110	15	2.5	30
CYP3A4 inhibitor	150	15	2.5	30
P-gp/CYP3A4 inducer	NR	NR	NR	30

BID, twice daily; OD, once daily; CrCl, creatinine clearance; P-gp, P-glycoprotein; CYP, cytochrome P450; NR, not recommended.

**Table 7:** Hypothesis-generating considerations for CCB dose individualization in selected patient populations and clinical scenarios based on the reported pharmacokinetic variability.

Drug	Elderly	Renal Impairment	DDI Risk	Recommendations
Amlodipine	No routine adjustment	No routine adjustment	No routine adjustment	Monitoring preferred
Diltiazem	Yes	No routine adjustment	Yes	Dose adjustment should be considered
Felodipine	No routine adjustment	No routine adjustment	No routine adjustment	Monitoring preferred
Isradipine	No evidence	Yes	Consider	Dose adjustment may be considered
Nicardipine	No evidence	No evidence	No evidence	Monitoring preferred
Nifedipine	No routine adjustment	Consider	Yes	Dose adjustment should be considered
Nimodipine	No evidence	No evidence	Yes	Dose adjustment should be considered
Nisoldipine	Yes	Yes	Yes	Dose adjustment should be considered
Verapamil	Consider	No evidence	Yes	Dose adjustment should be considered

Yes = supported by current evidence and/or product information; Consider = supported by PK variability findings, patient-specific assessment recommended; No routine adjustment = adjustment not generally recommended, although monitoring may be appropriate; No evidence = insufficient data to support a recommendation.

## Data Availability

The original contributions presented in this study are included in the article. Further inquiries can be directed to the corresponding authors.

## Supplementary Materials

The following supporting information can be downloaded at: https://www.mdpi.com/article/10.3390/pharmaceutics18091103/s1, Table S1. Descriptive statistics of pharmacokinetic variability for Cmax and AUC across single-dose and steady-state studies of DOACs and CCBs; Table S2. Statistically significant pairwise comparisons in PK variability between individual CCBs for Cmax and AUC; Table S3. Statistically significant pairwise comparisons of PK variability between study populations for Cmax and AUC among DOACs; Table S4. Overall model statistics and regression coefficients from multivariable analyses of PK variability; Figure S1. Population sources of PK variability and their impact on drug exposure variability among DOACs and CCBs: (a) mean PK variability, expressed as CV%, across different population subgroups for DOACs; and (b) mean PK variability (CV%) across population subgroups for CCBs.
