# Supplementary material for: Pharmacokinetic Variability of Direct Oral Anticoagulants and Calcium Channel Blockers: A Comparative Analysis of Exposure Data from Clinical Studies
Source: Pharmaceutics. 2026 Sep 2;18(9):1103. doi: 10.3390/pharmaceutics18091103 (PMC13610595; doi:10.3390/pharmaceutics18091103)
Supplement: Supplementary file 1 [file pharmaceutics-18-01103-s001.zip › pharmaceutics-4488422-supplementary.pdf]

Supplementary Materials: Pharmacokinetic Variability of Direct Oral Anticoagulants and Calcium Channel Blockers: A Comparative Analysis of Exposure Data from Clinical Studies

Lara Marques [1,2] and Nuno Vale [1,2,3,*]

Table S1. Descriptive statistics of pharmacokinetic variability for C_{max} and AUC across single-dose and steady-state studies of DOACs and CCBs.

Drug	Type of Study	Mean CV		SD CV		Median CV		IQR CV		Min CV		Max CV	
		C_{max}	AUC	C_{max}	AUC	C_{max}	AUC	C_{max}	AUC	C_{max}	AUC	C_{max}	AUC
**DOACs**													
Apixaban	Single-dose	27.94	22.37	13.44	17.16	27.94	23.5	12.26	29.55	2.30	0.39	72	82
	Steady-state	28.21	28.80	12.37	17.36	25.50	24	19.28	15.50	10	7	49.90	84
Rivaroxaban	Single-dose	25.42	26.23	11.52	13.38	26.93	26.77	11.98	14.83	0.57	0.09	57.10	59.10
	Steady-state	32.60	28.26	18.65	9.68	43.10	28.43	35.06	14.55	4.30	17.30	53.74	42.46
Edoxaban	Single-dose	31.80	21.04	9.30	7.90	29.90	20.29	10.74	7.53	12.70	8.10	51.30	45.16
	Steady-state	25.73	24.36	5.26	13.78	25.65	18	4.77	19.55	16.60	11.20	35.10	47
Dabigatran	Single-dose	34.88	30.64	24.57	19.32	29.69	26.35	25.57	26.09	4.65	4.55	123	79
	Steady-state	34.80	27.45	19.24	15.56	33	24.95	21.70	14.50	9.86	8.25	79.80	78.30
**CCBs**													
Amlodipine	Single-dose	23.06	34.07	8.89	35.66	21.38	28	6.95	7.63	2.20	2	56.39	344.81
	Steady-state	23.44	29.73	10.34	21.87	22.57	24.35	12.88	16.44	3	3.70	49.2	139.04
Felodipine	Single-dose	18.20	32.18	18.20	22.98	29.90	21.95	28.05	32.23	2.59	8.08	79	100.77
	Steady-state	31.35	29.79	14.88	14.27	32.99	34.56	21.05	20.41	6.40	6.80	50.70	50.18
Nifedipine	Single-dose	35.28	32.27	19.67	17.59	35.00	32.71	27.44	25.23	0.28	0.29	81.71	92.73
	Steady-state	33.99	26.59	23.77	21.53	39.08	31.17	45.67	39.09	1.48	0.14	71.68	73.52
Nicardipine	Single-dose	4.07	3.23	2.47	2.19	4.28	2.63	4.03	1.28	1.53	1.28	6.19	6.38
	Steady-state	61.68	-	7.32	-	60.80	-	6.63	-	53.80	-	71.30	-
Nisoldipine	Single-dose	84.06	81.42	47.59	50.45	68.75	59.10	51.05	40.27	42.11	41.30	174.53	177.46
	Steady-state	50.18	41.71	28.96	17.14	41.67	39.52	25.14	26.85	16.67	13.70	127.27	70.27
Isradipine	Single-dose	50.60	38.22	31.37	11.50	63.43	35.81	56.75	15.87	9.90	21.60	86.67	54.58
	Steady-state	53.92	55.99	8.92	2.89	52.40	56.78	8.83	2.81	45.85	52.79	63.50	58.41
Nimodipine	Single-dose	58.39	52.80	20.66	10.18	60.50	51.51	22.82	12.48	22.92	37.18	92.86	69.23
	Steady-state	11.47	15.86	11.31	19.17	6.95	7.65	10.10	12.83	2.19	3.05	38.90	60.34
Diltiazem	Single-dose	26.94	33.16	16.09	21.72	32	35.10	30.39	31.61	4.30	3.86	57	92
	Steady-state	29.90	31.90	14.90	15.73	33.19	36.07	20.57	22.32	6.80	5.70	59.11	63.61
Verapamil	Single-dose	39.87	35.30	23.39	20.60	37.80	31.24	20.15	32.67	8.28	11.04	86.49	70.27
	Steady-state	43.79	43.95	16.24	19.20	46.35	45.15	21.03	21.66	8.83	9.39	86.50	83.81

DOACs, direct oral anticoagulants; CCBs, calcium channel blockers; CV, coefficient of variation; SD, standard deviation; C_{max}, maximum plasma concentration; AUC, area under the plasma concentration–time curve; IQR, interquartile range; Min, minimum value; Max, maximum value. All CV values are expressed as percentages.

Table S2. Statistically significant pairwise comparisons in PK variability between individual CCBs for C_{max} and AUC.

PK Parameter	Study Design	Test Comparison	Z	Adjusted p-value
C_{max}	SD	Amlodipine–Nifedipine	3.88	0.0034**
		Amlodipine–Nimodipine	4.62	0.0001***
		Amlodipine–Nisoldipine	4.50	0.0002***
		Diltiazem–Nimodipine	3.75	0.0052**
		Diltiazem–Nisoldipine	3.80	0.0045**
		Felodipine–Nimodipine	3.62	0.0075**
		Felodipine–Nisoldipine	3.67	0.0067**
		Isradipine–Nicardipine	–3.59	0.0082**
		Nicardipine–Nifedipine	3.70	0.0063**
		Nicardipine–Nimodipine	4.77	<0.001***
		Nicardipine–Nisoldipine	4.86	<0.001***
		Nicardipine–Verapamil	3.66	0.0068**
	SS	Amlodipine–Nicardipine	3.89	0.0031**
		Amlodipine–Nisoldipine	3.99	0.0021**
		Amlodipine–Verapamil	5.85	<0.001***
		Diltiazem–Verapamil	3.61	0.0091**
		Isradipine–Nimodipine	–3.55	0.0110*
		Nicardipine–Nimodipine	–4.39	0.0003***
		Nifedipine–Nimodipine	–3.30	0.0267*
		Nimodipine–Nisoldipine	4.23	<0.0007***
		Nimodipine–Verapamil	4.88	<0.001***
AUC	SD	Amlodipine–Nimodipine	3.98	0.0023*
		Amlodipine–Nisoldipine	3.83	0.0041**
		Diltiazem–Nicardipine	–3.16	0.0373*
		Diltiazem–Nimodipine	3.31	0.0264*
		Diltiazem–Nisoldipine	3.28	0.0277*
		Felodipine–Nimodipine	3.85	0.0039**
		Felodipine–Nisoldipine	3.73	0.0058**
		Isradipine–Nicardipine	–3.45	0.0171*
		Nicardipine–Nifedipine	3.26	0.0277*
		Nicardipine–Nimodipine	4.76	0.0001****
		Nicardipine–Nisoldipine	4.77	0.0001****
		Nifedipine–Nimodipine	3.32	0.0259*
		Nifedipine–Nisoldipine	3.38	0.0277*
	SS	Amlodipine–Verapamil	4.30	0.0004***
		Isradipine–Nimodipine	–3.33	0.0216*
		Nifedipine–Verapamil	3.80	0.0037**
		Nimodipine–Nisoldipine	3.28	0.0248*

	Nimodipine–Verapamil	3.94	0.0022**

AUC, area under the plasma concentration–time curve; C_{max}, maximum plasma concentration; SD, single-dose; SS, steady-state; Z, standardized test statistic. Adjusted *p*-values were obtained using Dunn's post-hoc test with Holm correction for multiple comparisons. Only statistically significant pairwise comparisons are presented. $p < 0.05$; $p < 0.01$; $p < 0.001$.

Table S3. Statistically significant pairwise comparisons of PK variability between study populations for C_{max} and AUC among DOACs.

PK Parameter	Test Comparison	Z	Adjusted p-value
C_{max}	Elderly–Other	4.03	0.0025**
AUC	DDI–Elderly	–3.77	0.0072**

AUC, area under the plasma concentration–time curve; C_{max}, maximum plasma concentration; Z, standardized test statistic. Comparisons were performed using Dunn's post-hoc test following the Kruskal-Wallis test. Adjusted *p*-values account for multiple comparisons with Holm correction. Only statistically significant pairwise comparisons are presented. $p < 0.01$.

Table S4. Overall model statistics and regression coefficients from multivariable analyses of PK variability.

**Overall model statistics**				
**Model**	**F-statistic**	**p-value**	**R^2**	**Adjusted R^2**
DOAC C_{max}	2.92	<0.001	0.101	0.067
DOAC AUC	3.97	<0.001	0.145	0.109
CCB C_{max}	8.50	<0.001	0.199	0.176
CCB AUC	8.07	<0.001	0.197	0.172

**Regression coefficients**					
**Model**	**Variable**	**β**	**SE**	**exp(β)**	***p*-value**
DOAC C_{max}	**Drug**				
	Dabigatran	0.201	0.130	1.223	0.123
	Edoxaban	0.129	0.154	1.138	0.404
	Rivaroxaban	–0.126	0.128	0.882	0.329
	**Type of Study**				
	Steady-state	–0.015	0.095	0.985	0.870
	**Population**				
	DDI	0.022	0.143	1.022	0.879
	Elderly	–0.896	0.261	0.408	<0.001***
	Ethnicity	–0.227	0.139	0.797	0.104
	Hepatic Impairment	0.277	0.305	1.319	0.364
	Other	0.258	0.175	1.294	0.141
	Pediatric	–0.015	0.246	0.985	0.950
	Renal Impairment	–0.027	0.162	0.973	0.866
	Sex	0.052	0.202	1.053	0.796
DOAC AUC	**Drug**				
	Dabigatran	0.680	0.200	1.974	<0.001***

	Edoxaban	0.223	0.250	1.250	0.373
	Rivaroxaban	0.455	0.194	1.576	0.020*
	**Type of Study**				
	Steady-state	0.141	0.163	1.151	0.390
	**Population**				
	DDI	–0.056	0.220	0.946	0.800
	Elderly	–1.654	0.383	0.191	<0.001***
	Ethnicity	–0.642	0.208	0.526	0.002**
	Hepatic Impairment	–0.639	0.446	0.528	0.153
	Other	–0.220	0.285	0.803	0.441
	Pediatric	–0.087	0.374	0.917	0.816
	Renal Impairment	–0.057	0.273	0.945	0.834
	Sex	–0.121	0.334	0.886	0.718
CCB C_{max}	**Drug**				
	Diltiazem	0.380	0.113	1.462	<0.001***
	Felodipine	0.329	0.103	1.390	0.001**
	Isradipine	0.809	0.236	2.246	<0.001***
	Nicardipine	0.137	0.279	1.147	0.623
	Nifedipine	0.183	0.096	1.201	0.057
	Nimodipine	0.248	0.185	1.281	0.180
	Nisoldipine	1.072	0.174	2.921	<0.001***
	Verapamil	1.007	0.129	2.737	<0.001***
	**Type of Study**				
	Steady-state	–0.018	0.070	0.982	0.791
	**Population**				
	DDI	0.466	0.097	1.594	<0.001***
	Elderly	0.001	0.129	1.001	0.996
	Ethnicity	0.497	0.122	1.644	<0.001***
	Hepatic Impairment	0.738	0.442	2.092	0.095
	Other	–0.340	0.115	0.712	0.003**
	Renal Impairment	–0.001	0.152	0.999	0.996
	Sex	0.533	0.300	1.704	0.073
CCB AUC	**Drug**				
	Diltiazem	0.357	0.140	1.429	0.011*
	Felodipine	0.105	0.134	1.111	0.432
	Isradipine	0.567	0.296	1.763	0.056
	Nicardipine	–1.741	0.491	0.175	<0.001***
	Nifedipine	–0.466	0.121	0.628	<0.001***
	Nimodipine	0.130	0.236	1.139	0.583
	Nisoldipine	0.782	0.271	2.186	<0.001***

Verapamil	0.879	0.171	2.408	<0.001***
**Type of Study**				
Steady-state	–0.276	0.090	0.759	0.002**
**Population**				
DDI	0.513	0.124	1.670	<0.001***
Elderly	0.136	0.169	1.146	0.422
Ethnicity	0.618	0.153	1.855	<0.001***
Hepatic Impairment	0.701	0.565	2.016	0.216
Other	–0.224	0.151	0.799	0.139
Renal Impairment	–0.031	0.186	0.969	0.866
Sex	0.513	0.372	1.670	0.169

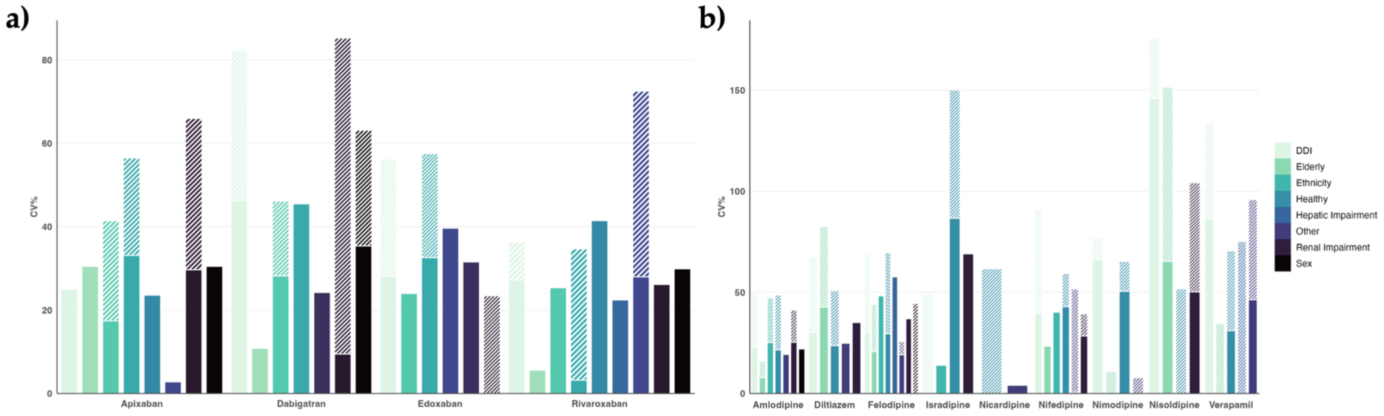

Figure S1. Population sources of PK variability and their impact on drug exposure variability among DOACs and CCBs: (a) mean PK variability, expressed as CV%, across different population subgroups for DOACs; and (b) mean PK variability (CV%) across population subgroups for CCBs.
